# Persistent Pulmonary Hypertension of the Newborn: Pathophysiological Mechanisms and Novel Therapeutic Approaches

**DOI:** 10.3389/fped.2020.00342

**Published:** 2020-07-24

**Authors:** Sofia Martinho, Rui Adão, Adelino F. Leite-Moreira, Carmen Brás-Silva

**Affiliations:** ^1^Department of Surgery and Physiology, Cardiovascular Research and Development Center—UnIC, Faculty of Medicine, University of Porto, Porto, Portugal; ^2^Faculty of Nutrition and Food Sciences, University of Porto, Porto, Portugal

**Keywords:** persistent pulmonary hypertension of the newborn, pulmonary vasoconstriction, pulmonary vascular remodeling, pulmonary vascular resistance, pulmonary vasodilators

## Abstract

Persistent pulmonary hypertension of the newborn (PPHN) is one of the main causes of neonatal morbidity and mortality. It is characterized by sustained elevation of pulmonary vascular resistance (PVR), preventing an increase in pulmonary blood flow after birth. The affected neonates fail to establish blood oxygenation, precipitating severe respiratory distress, hypoxemia, and eventually death. Inhaled nitric oxide (iNO), the only approved pulmonary vasodilator for PPHN, constitutes, alongside supportive therapy, the basis of its treatment. However, nearly 40% of infants are iNO resistant. The cornerstones of increased PVR in PPHN are pulmonary vasoconstriction and vascular remodeling. A better understanding of PPHN pathophysiology may enlighten targeted and more effective therapies. Sildenafil, prostaglandins, milrinone, and bosentan, acting as vasodilators, besides glucocorticoids, playing a role on reducing inflammation, have all shown potential beneficial effects on newborns with PPHN. Furthermore, experimental evidence in PPHN animal models supports prospective use of emergent therapies, such as soluble guanylyl cyclase (sGC) activators/stimulators, l-citrulline, Rho-kinase inhibitors, peroxisome proliferator-activated receptor-γ (PPAR-γ) agonists, recombinant superoxide dismutase (rhSOD), tetrahydrobiopterin (BH4) analogs, ω-3 long-chain polyunsaturated fatty acids (LC-PUFAs), 5-HT2A receptor antagonists, and recombinant human vascular endothelial growth factor (rhVEGF). This review focuses on current knowledge on alternative and novel pathways involved in PPHN pathogenesis, as well as recent progress regarding experimental and clinical evidence on potential therapeutic approaches for PPHN.

## Introduction

Persistent pulmonary hypertension of the newborn (PPHN) is characterized by sustained elevation of pulmonary vascular resistance (PVR), caused by a failure in the circulatory adaptation that normally occurs within minutes after delivery. This leads to right-to-left shunting of blood through *foramen ovale* and *ductus arteriosus* and prevents the increase in pulmonary blood flow (PBF), essential for extrauterine oxygenation and survival (1). Therefore, PPHN usually presents shortly after birth, precipitating severe respiratory distress and hypoxemia. Newborns with PPHN are at high risk of severe asphyxia and its complications, including neurological injury, multiorgan dysfunction, and death (2).

The incidence of PPHN is ~2/1,000 live births (3, 4), being highest in term and late preterm infants (4). Despite advances in neonatal cardiorespiratory care, PPHN is still one of the main causes of neonatal morbidity and mortality, with a mortality rate of 4–33% (3).

PPHN is associated with multiple possible etiologies, usually accountable for its severe outcomes (5). Although a life-threatening condition, it can, in most cases, be reversible within the first days of life, alongside the improvement of the associated etiology. However, the heterogeneity of etiopathogenic factors prevents the adoption of a standardized treatment, leading to challenges in its management (6). Besides approved nitric oxide, treatment is limited to the experimental use of new drugs or those approved for pulmonary arterial hypertension (PAH) in adults (6), despite distinct pathogenesis.

A better understanding of PPHN pathophysiology may enlighten targeted and more effective therapies. Hence, this review will focus on current knowledge on alternative and novel etiopathogenic pathways, as well as recent progress regarding experimental or clinical evidence on potential therapeutic options for PPHN, particularly if resistant to current treatment. Emphasis will be PPHN in general, mostly without addressing pathogenic mechanisms involved in specific etiologies. However, when relevant, some considerations will be made regarding several associated conditions.

## Methods for Literature Search

We identified references for this review by doing a PubMed search through the time period 1980 to present. Without any language restriction, we used the following search terms in combination with the terms “pulmonary hypertension of newborn” or “persistent fetal circulation syndrome”: “mechanisms,” “etiology,” “physiopathology,” “experimental models,” “epidemiology,” “guidelines,” “classification,” “therapy,” “treatment,” and “vasodilators.” We also searched for “Persistent Pulmonary Hypertension of the Newborn” in Cochrane Library and ClinicalTrials.gov. The last search was conducted in January 2020. Additional articles were identified by cross-referencing from author reference lists and published review papers. The two authors SM and RA independently assessed the titles and abstracts for suitability to be included in this review. We obtained the full-text version of identified papers and settled any differences by discussion.

## Transition of the Pulmonary Circulation at Birth

A succession of cardiopulmonary adaptations are expected to occur at birth, allowing a smooth transition from fetal to extrauterine life. The removal of the low-resistance placental circulation, with clamping of the umbilical cord, increases systemic vascular resistance, leading to a rapid increase in arterial pressure and reduction in cardiac output (7). Concurrently, a series of orchestrated physiological events are involved in the rapid PVR decrease and consequent 8- to 10-fold increase in PBF, determinant for the establishment of gas exchange after birth. Although the precise mechanisms are still unclear, the resultant cardiovascular transformations from increased PBF are well-established (7). Flow reversal across the *ductus arteriosus*, as well as closure of the *foramen ovale*, is expected, shifting the cardiovascular configuration from parallel, in the fetus, to in series, leading to equal left and right ventricular output in the newborn (8). This does not occur in the setting of persistently elevated PVR (PPHN), in which right-to-left shunting persists (1).

High fetal PVR, responsible for diverting most of the right ventricular output away from the liquid-filled lungs, results primarily from low oxygen tension, due to pulmonary vasoconstrictor response to hypoxia and low PBF (9). These determine suppression of nitric oxide (NO) and prostacyclin (PGI2) production and release from the pulmonary endothelium, as well as increased levels of vasoconstrictors [endothelin-1 (ET-1), thromboxane (TXA2), and leukotrienes] and altered smooth muscle cell reactivity. Furthermore, the fluid-filled airspace likely contributes to high PVR, by creating high vascular extraluminal pressure (10). However, in late gestation, the pulmonary vasculature acquires the ability to respond to vasodilator stimuli, through maturational changes in pulmonary artery endothelial cells (PAECs) and pulmonary artery smooth muscle cells (PASMCs), vital for enabling successful transition at birth (10).

After delivery, the PVR declines dramatically with the first postnatal breath. This results from the combined effect of increased alveolar oxygen tension and the onset of ventilation itself, with lung distention and aeration (11). These physical/mechanical stimuli, including the increased shear stress from augmented PBF, cause pulmonary vasodilation in part by increasing production of vasodilators, such as NO and PGI2 (10).

Oxygen has long been recognized as one of the most important stimuli for perinatal pulmonary vasodilation. Increased PaO_2_ alone, in the absence of ventilation, can, in animal models, reproduce the pulmonary vasodilation seen at birth (12). Oxygenation has many effects, including relaxation of PASMCs through interference on NO–cyclic guanylyl monophosphate (NO-cGMP) and prostacyclin–cyclic adenosine monophosphate (PGI2-cAMP) pathways (1), as later discussed in this review. Thus, decreased ability to produce these mediators in the setting of a perinatal stress, such as asphyxia, may result in persistently increased PVR, thus PPHN.

Likewise, shear stress, besides prompting increased release of NO and PGI2, also promotes structural reorganization of the pulmonary vessels, with flattening of the endothelium and thinning of PASMCs and the matrix, further promoting vasodilation (10, 13).

Effective clearance of fetal lung fluid, usually completed in the first few hours of life, also contributes for the decrease in PVR and is estimated to be responsible for a 3- to 4-fold increase in PBF by itself (14). Fluid clearance starts with onset of labor (or even before), with increased cortisol, catecholamines, and thyroid hormone levels stimulating active fluid absorption by the respiratory epithelium, through active sodium transport (9, 15). Moreover, transepithelial pressure gradients generated by inspiration further contribute to the airway liquid clearance after birth (7). In fact, even during intrauterine life, PBF markedly increases (up to 4-fold) during periods of accentuated fetal breathing movements (16). Finally, changes in transpulmonary pressure due to alterations in fetal posture, resultant from uterine contractions during labor, also seem to play a role on forcing liquid out of the lungs (15). Failure to clear lung fluid occurs in transient tachypnea of newborn, particularly after elective cesarean delivery, which is one of the etiologies associated with PPHN (5).

On the other hand, ventilation alone, without changing oxygenation, has been increasingly recognized as a major stimuli for decreasing PVR at birth and has shown to experimentally increase PBF by 400% (9). Thus, besides increased oxygenation, other mechanisms are also involved in the ventilation-induced increase in PBF, such as augmented lung recoil due to establishment of an air/liquid interface and the development of surface tension within the lung (14).

Based on the previously discussed mechanisms, such as increased oxygen and shear stress, with subsequent increased local release of NO and PGI2, it would be expected that increased ventilation would act locally to dilate adjacent blood vessels. However, increase in PBF has been experimentally shown not to be spatially related to ventilated lung regions, since partial lung aeration is able to cause a rapid and simultaneous global increase in PBF (17). Furthermore, this effect is independent of changes in inspired oxygen, since partial lung aeration with 100% nitrogen mimics the global increase in PBF (18). However, aeration with 100% oxygen leads to enhanced PBF increase specifically in ventilated regions, underscoring oxygenation as a stimuli to localized increase in PBF (18).

Thus, neither a spatial relationship nor increased oxygenation can fully explain the global decrease in PVR following onset of ventilation. A parasympathetic neural response has been recently suggested as the mediator for this association. In fact, bilateral *sectioning* of the vagus nerves hampers the described global vasodilating response, despite not interfering with the localized enhancing effect of oxygenation (19). This reflex might be mediated by an afferent stimulation of vagus nerve's C fibers by increased pressure within perialveolar tissue, due to clearance of the fetal lung liquid (19).

These observations challenge previous understanding on the critical decrease in PVR at birth, contesting the role of oxygen as the dominant factor in this event. Instead, they emphasize the importance of lung aeration, at least in part through vagal stimulation, and underline the importance of, above all, establishing effective ventilation during newborn resuscitation (20). Moreover, modulation of these novel mechanisms may open doors to potential targeted therapies for PPHN, such as enhancement of parasympathetic activation, which could contribute to decreasing PVR in these newborns.

Furthermore, the additive effects of oxygenation, not mediated by this mechanism, emphasize that multiple, overlapping, interrelated, and synchronized stimuli coordinate to ensure the increase in PBF after birth. Therefore, there are specific mediators that facilitate cardiovascular transition, such as the vasodilators NO and PGI_2_, whose pathways might be of interest as targeted therapies for PPHN, as discussed in this review. However, pulmonary circulatory transition is a complex process involving the coordinated regulation of many physiological events, which in part contribute for stimulating the release of these mediators, with the ultimate goal of decreasing PVR. If one of these concerted mechanisms is, in some way, defective, due to a wide variety of possible etiologies (5), newborns might fail to achieve or sustain the normal decline in PVR and PPHN may result.

## Pathogenesis of PPHN

Two essential mechanisms may be involved in the basis of PPHN pathogenesis, both resulting in increased PVR after birth: increased pulmonary arteriolar vasoconstriction and vascular structural remodeling, with or without underdevelopment of pulmonary vascular bed. However, many different perinatal disorders might be involved in causing both these phenomena and are recognized as PPHN etiologies, as described in Table 1 (5, 6).

**Table 1:** Persistent pulmonary hypertension of the newborn (PPHN) and its multiple associated etiologies (5, 6).

**PPHN associated neonatal disorders**	
**Idiopathic PPHN** ◦Normal pulmonary parenchyma with abnormally remodeled pulmonary vasculature ◦10-20% of all PPHN cases	
**Lung parenchymal diseases** ◦Abnormally constricted pulmonary vasculature	Meconium aspiration syndrome (MAS)
	Pneumonia/sepsis
	Respiratory distress syndrome (RDS)^*^
**Abnormal transition at birth** ◦Impaired pulmonary vasodilation	Transient tachypnea of the newborn (TTN)
	Perinatal stress/asphyxia
**Developmental lung disease** ◦Hypoplastic pulmonary vasculature	Congenital diaphragmatic hernia (CDH)
	Oligohydramnios
	Down syndrome

* *RDS can be considered as both a lung parenchymal disease and an expression of an abnormal transition at birth*.

Therefore, the pathogenesis of PPHN is multifactorial, also involving several perinatal risk factors such as maternal exposures (4, 21–23), as described in Table 2. The specific mechanism linking these factors and PPHN remains unclear for most of them (6). However, independent of causality, these factors predict higher risk and should raise clinicians' awareness for PPHN.

**Table 2:** Perinatal risk factors that predict higher risk for persistent pulmonary hypertension of the newborn (PPHN) (4, 21–23).

**Risk factors for PPHN**
•**Perinatal exposures** ◦ Maternal SSRIs intake ◦ Maternal NSAIDs intake ◦ Mode of delivery: highest in cesarean section ◦ Maternal diabetes and overweight ◦ Maternal smoking ◦ Maternal asthma (inconsistent evidence)
•**Intrinsic characteristics of the newborn** ◦ Maternal ethnicity: highest in black race, lowest in Hispanic ethnicity ◦ Gestational age: highest in late preterm (and early term) newborns ◦ Birth weight: highest in small and large for gestational age ◦ Gender: highest in male sex

*SSRIs, selective serotonin reuptake inhibitors; NSAIDs, non-steroidal anti-inflammatory drugs*.

### Pulmonary Vasoconstriction and Vascular Remodeling: The Cornerstones of PPHN

Increased vasoconstriction and vascular remodeling can both be primary or secondary to the previously described etiologies. They may be found together, playing a more or less preponderant role on increasing PVR, depending on the underlying etiopathogenic factors (24).

Augmented vasoconstriction results from an imbalance between vasoconstrictor and vasodilating agents, mostly produced by PAECs, favoring increased PASMCs contraction, hence increased vascular tone. Perinatal asphyxia and sepsis are two examples of etiologies leading to markedly increased pulmonary arteriolar vasoconstriction, either directly, through hypoxia and acidosis, or indirectly, via release of vasoconstrictor substances, such as ET-1, TXA2, and leukotrienes (25).

Increased vascular remodeling results from PASMCs' disturbed development and enhanced proliferation, leading to hyperplasia and hypertrophy of vascular smooth muscle layer, narrowing vascular lumen, and increasing PVR. This is mainly, but not exclusively, observed in cases of idiopathic PPHN, such as newborns exposed to chronic intrauterine hypoxia or increased fetal pulmonary arterial pressure (PAP) due to, for example, premature closure of *ductus arteriosus* (26).

In fact, many signaling pathways are pathogenic via both of these routes, such is the case of increased ET-1, serotonin, reactive oxygen species, and Rho-kinase, as will be discussed.

Most of the preclinical evidence disclosed in this review resulted from research in a lamb model of PPHN, which mimics the physiopathological characteristics associated to human PPHN, including augmented vascular constriction and smooth muscle hypertrophy, leading to persistently increased PVR, PAP, and right ventricular (RV) hypertrophy.

## Current Therapy for PPHN

Treatment of PPHN depends on the underlying disorder, aiming to decrease PVR and reduce the magnitude of the right-to-left shunt, mainly by administering pulmonary vasodilators (6). Depending on the severity, some infants may also require aggressive support of cardiac function, systemic blood pressure, and perfusion. Ventilation is crucial for improving ventilation/perfusion matching, and oxygen, although a recognized pulmonary vasodilator, hence many times used in high concentrations in these infants, may also be deleterious (6). If such treatment fails, with persisting severe respiratory failure, extracorporeal membrane oxygenation (ECMO) is required as a bridge therapy in the management of newborns with PPHN (6).

**Figure 1:**
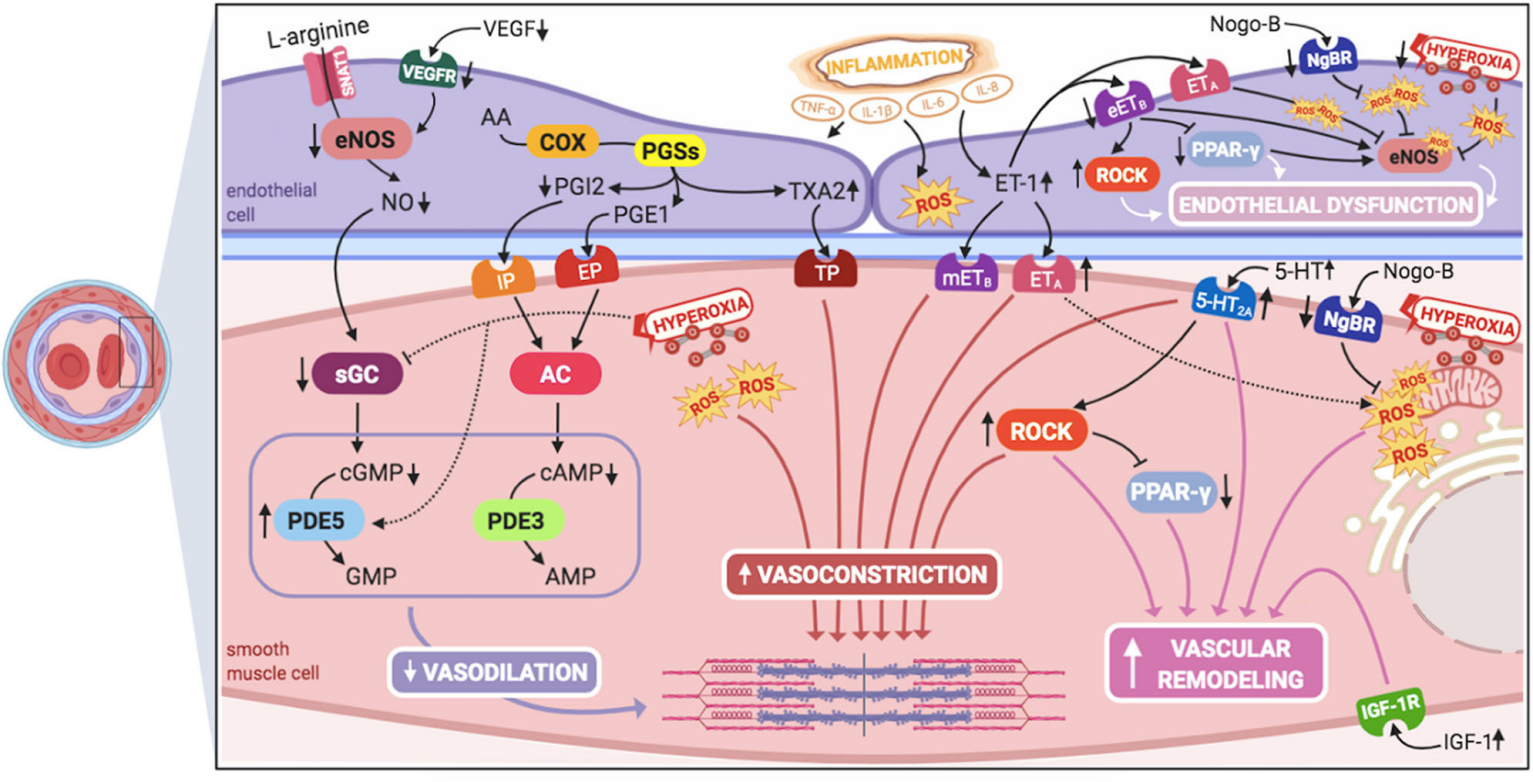
Signaling pathways involved in the pathogenesis of persistent pulmonary hypertension of the newborn (PPHN) and its interactions. 5-HT, serotonin; 5-HT_2A_, 5-HT receptor 2A; AA, arachidonic acid; AC, adenylyl cyclase; AMP, adenosine monophosphate; cAMP, cyclic adenosine monophosphate; cGMP, cyclic guanylyl monophosphate; COX, cyclooxygenase; eETB, endothelial relaxant endothelin receptor B; eNOS, nitric oxide synthase; EP, PGE1-receptor; ET-1, endothelin-1; ETA, endothelin receptor A; GMP, guanylyl monophosphate; IGF-1, insulin growth factor 1; IGF-1R, IGF-1 receptor; IL-1β, IL-6, IL-8, interleukins-1β, 6, and 8; IP, PGI2-receptor; mETB, smooth muscle contractile endothelin receptor B; NgBR, Nogo-B receptor; NO, nitric oxide; PDE3, phosphodiesterase-3; PDE5, phosphodiesterase-5; PGE1, prostaglandin E1; PGI2, prostacyclin; PGSs, prostaglandin synthases; PPAR- γ, peroxisome proliferator-activated receptor-γ; ROCK, Rho-kinase; ROS, reactive oxygen species; sGC, soluble guanylyl cyclase; SNAT1, sodium-coupled neutral amino acid transporter; TNF-α: tumor necrosis factor α; TP, TXA2-receptor; TXA2, thromboxane A2; VEGF, vascular endothelial growth factor; VEGFR, VEGF receptor. This figure was created with BioRender.com.

Inhaled nitric oxide (iNO) is the only approved pulmonary vasodilator specifically for the treatment of PPHN, although it does not improve survival and ~40% of neonates fail to respond to it (6, 27). Therefore, knowledge on different pathophysiological pathways, which might serve as potential therapeutic targets for the treatment of iNO-resistant PPHN (Figure 1), might be the key for ameliorating outcomes and improving survival of these infants.

## Pathophysiology and Targeted Therapeutic Approaches

### Nitric Oxide–cGMP Pathway

#### Pathway

**Figure 2:**
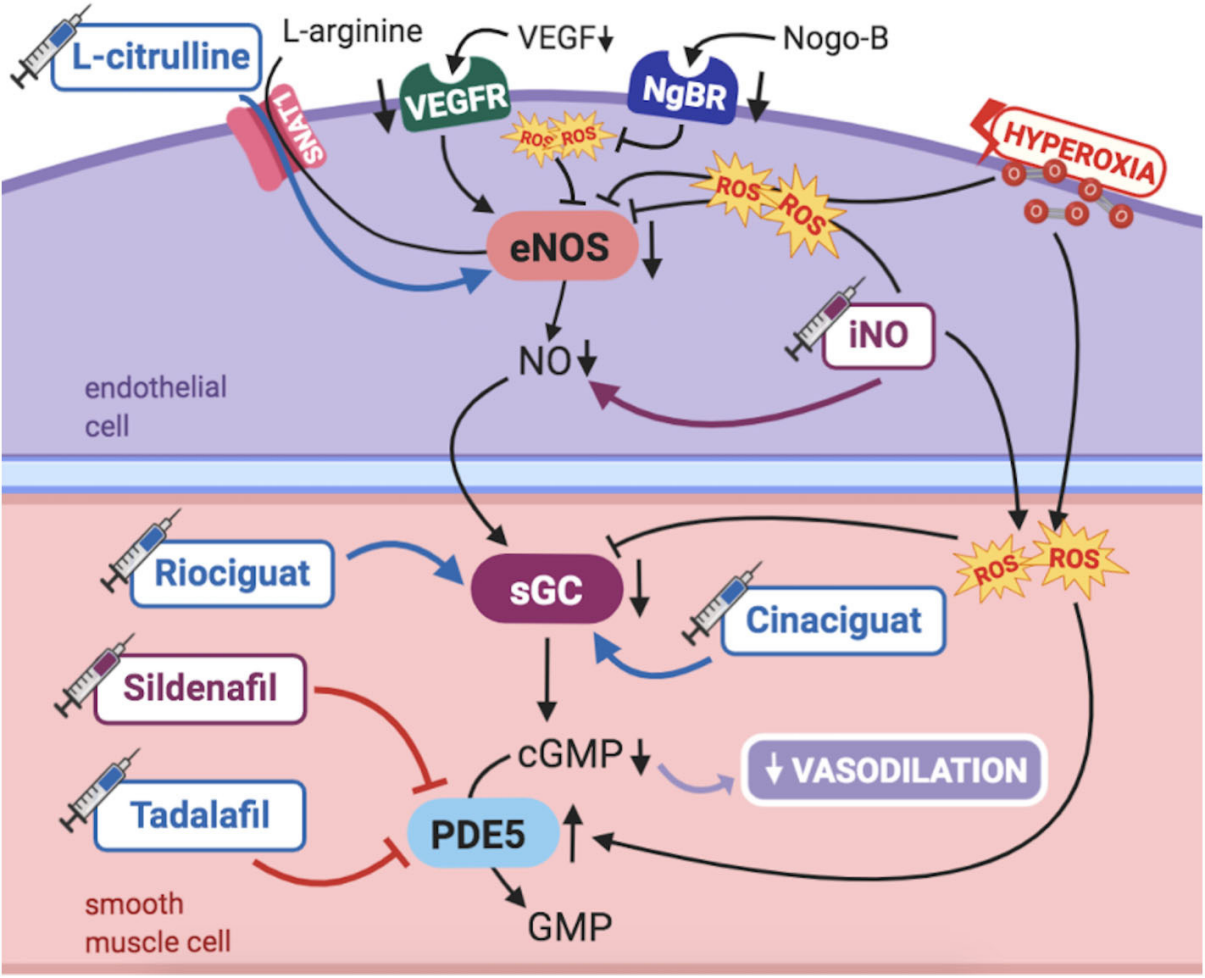
Pathogenic mechanisms of persistent pulmonary hypertension of the newborn (PPHN) and its current and potential target therapies: NO-cGMP pathway. cGMP, cyclic guanylyl monophosphate; eNOS, nitric oxide synthase; GMP, guanylyl monophosphate; iNO, inhaled nitric oxide; NgBR, Nogo-B receptor; NO, nitric oxide; PDE5, phosphodiesterase-5; ROS, reactive oxygen species; sGC, soluble guanylyl cyclase; SNAT1, sodium-coupled neutral amino acid transporter; VEGF, vascular endothelial growth factor; VEGFR, VEGF receptor. Target therapies are marked with a syringe icon and are colored based on the type of evidence supporting its use on PPHN—purple, evidence on its was obtained by adequately powered RCTs/meta-analysis; pink, evidence limited to observational studies or small and underpowered RCTs and/or inconsistent results in human newborns; blue, beneficial effects only demonstrated in experimental models of PPHN. This figure was created with BioRender.com.

Studies in humans and animal models have long been demonstrating the maturational changes occurring in the fetal pulmonary vasculature to adapt to transitional stimulus, mainly increased PaO_2_. PAECs release numerous vasoactive substances, such as nitric oxide (NO), essential for pulmonary transition at birth. Thus, NO-cGMP pathway has been a topic of particular interest in PPHN pathogenesis (Figure 2).

NO is produced by the endothelial nitric oxide synthase (eNOS) in PAECs from the substrate l-arginine, then diffusing to PASMCs. It then activates soluble guanylyl cyclase (sGC), triggering the production of cGMP (1), which ultimately leads to vasodilation (1). Phosphodiesterase 5 (PDE5) is the enzyme responsible for breaking down cGMP in PASMCs. Accordingly, its inhibition leads to further vasodilation. As such, vascular responsiveness to endogenous or exogenous NO is dependent upon the activity of all enzymes and intermediate targets comprised in the NO-cGMP pathway (1).

NO production is modulated through eNOS expression and activity, which are affected by multiple factors including oxygen tension, shear stress, paracrine factors [like vascular endothelial growth factor (VEGF)], superoxide (which inactivates NO and uncouples eNOS), as well as l-arginine (substrate) and tetrahydrobiopterin (BH4) (cofactor) availability (1). Under certain conditions, such as substrate or cofactor unavailability, eNOS uncouples, further contributing to increased reactive oxygen species (ROS) generation and an oxidative stress milieu, with its own role on pulmonary vasoconstriction (28).

Several mechanisms contribute to progressive changes in NO-cGMP pathway during fetal and early postnatal life, interfering with pulmonary vasoreactivity. In infants with PPHN, one or several of the steps in this signaling may be altered. First, NO production greatly increases in late gestation and early postnatal period, most likely due to increased oxygen tension, besides the increased sensitivity of PASMCs to relax in response to NO (1). NO production in PAECs is enhanced through increased eNOS expression and activity, which normally occurs at term gestation (29). eNOS decreased activity reduces NO levels and induces pulmonary vasoconstriction, resulting in PPHN in newborn lambs (30, 31). Similarly, sGC levels are highest in late gestation, while lung PDE5 activity is markedly elevated during fetal life, rapidly falling at birth. Thus, PDE5 activity appears to be fundamental in the maintenance of PVR *in utero* and in its striking immediate postnatal decrease (1). All these findings have supported, throughout the years, the widely acknowledged major role of NO-cGMP signaling pathway on the modulation of fetal pulmonary circulation and its transition to extrauterine life.

#### Potential Targeted Therapies

##### Inhaled nitric oxide

iNO is the most obvious, as well as the most studied and accepted treatment of PPHN, acting locally as pulmonary vasodilator in PASMCs. In 2017, a meta-analysis including 17 randomized controlled trials (RCTs) compared iNO use with control, in term or late preterm newborns with PPHN (32). No significant difference was found in mortality, although there was a significant reduction in the need for ECMO. Besides, oxygenation was significantly improved, with decreased risk of neurodevelopmental sequelae and pulmonary complications (32). However, benefits of iNO are not clear when pulmonary hypertension (PH) is associated to congenital diaphragmatic hernia (CDH) (32). Furthermore, iNO has been proven safe to use in neonates, without systemic adverse events or long-term use toxicity. As such, iNO is considered effective and safe for use in infants with PPHN, except for those with associated CDH (32), as summarized in Table 3. However, besides not reducing PPHN mortality and being a costly intervention, ~40% of neonates with PPHN fail to respond to iNO, with some of those further experiencing a rebound PH, most likely due to suppression of endogenous NO production (47).

**Table 3:** An overview of therapeutic approaches for PPHN whose use has been studied in human infants.

**Treatment options**		**Mechanism of action**	**Type of evidence**	**Efficacy**	**Dosage schedule and clinically relevant observations**
iNO		Exogenous NO	Meta-analysis (including 17 RCTs) (32)	•Decreased need for ECMO: RR = 0.60 (0.50–0.71) •Improved OI: MD = −8.45 (−11.42 to −5.48) •Improved PaO_2_: MD = 32.62 mmHg (23.56–41.67)	•Dose: 10–80 ppm (inhaled) •iNO initiated if OI ≥ 25 or PaO_2_ <100 mmHg with 100% O_2_ •Quality of evidence: high
Sildenafil		PDE5 inhibitor	Meta-analysis (including 5 RCTs) (33)	•Mortality reduction: RR = 0.20 (0.07–0.56)	•Dose: 0.5–3.0 mg/kg every 6 h + variable loading doses (IV, oral or inhaled) •Quality of evidence: low
PGI2 analogues	Iloprost	Exogenous PGI2	Case series (33 infants) (34)	•Improved OI, PaO_2_, and SpO_2_ (*p* <0.05) •Improved AaDO_2_: MD = −24 mmHg (*p* = 0.02)	•Dose: 110 ng/kg/min, for a median duration of 97 h (11–480 h) (IV) •Decreased systemic blood pressure
			Observational: iloprost vs. sildenafil (20 infants received iloprost) (35)	•Decreased duration of MV: MD = −3.8 days (*p* < 0.001) •Improved PAP (*p* < 0.05) and AaDO_2_ (*p* < 0.001) •Shorter time to an adequate response (*p* < 0.03)	•Dose: 1–2.5 mg/kg every 2–4 h, for a mean duration of 5.41 ± 2.79 days (inhaled) •No systemic hypotension
	Epoprostenol		Case series (8 infants) (36)	•Decreased PAP: MD = −19.4 mmHg (*p <* 0.0005)	•Dose: 20 ng/kg/min, increased to a mean dose of 60 ng/kg/min, for a median duration of 3.6 days (IV) •No systemic hypotension
			Case series (4 preterm infants) (37)	•Improved OI: MD = −32 (39 ± 13.3 to 7 ± 2.5) •Improved PaO_2_/FiO_2_: MD = 171 (47 ± 13.0 to 218 ± 66.6)	•Dose: 50 ng/kg (endotracheal single bolus) •No systemic hypotension
	Treprostinil		Case reports (2 preterm infants) (38)	•Substantial clinical improvement in the first hours after treatment	•Dose: 5 ng/kg/min, titrated to a max. of 20 ng/kg/min, for 5 or 7 days (IV) •No apparent systemic adverse effects, including intraventricular hemorrhage
	Beraprost		Case series (7 infants) (39)	•Improved OI: MD = −18.2 (*p* = 0.018)	•Dose: 1 mcg/kg every 6 h, for a median duration of 101 h (40–205 h) (oral) •Decreased systemic blood pressure
Alprostadil		Exogenous PGE1	Case series (9 infants) (40)	•Shortened length of stay: MD = 11 days (*p* = 0.004)	•Dose: 20 (5–100) ng/kg/min (IV) •No need for higher rates of ventilation or inotrope use
Milrinone		PDE3 inhibitor	Case series(9 infants) (41),(11 infants) (42)	•Improved OI: MD = −8.00 (−14.6 to −1.4) (*p* < 0.05)	•Dose: 0.33–0.99 mcg/kg/min, for a median duration of 70 h (23–136 h) (IV) •No systemic hypotension
				•Improved PaO_2_: MD = 31 mmHg (*p* = 0.002) •Improved OI: MR = 65% (6–87%) (*p* < 0.001) •Improved echocardiographic indices: lower PAP, improved RV + LV output and reduced shunting (*p* < 0.05)	•Dose: 50 mcg/kg (loading dose) + 0.33 mcg/kg/min, titrated to a max. of 1.4mg/kg/day, for a median duration of 24 h (24-42 h) (IV) •No systemic hypotension or intraventricular hemorrhage
Bosentan		ET-receptors non-selective antagonist	Case series (40 infants) (43)	•Improved OI (*p* = 0.002) and AaDO_2_ (*p* = 0.01) •Improved SpO_2_: MD = 5% (*p* < 0.001)	•Dose: 1 mg/kg b.i.d, for a mean duration of 6.2 ± 3.1 days (oral) •No hepatotoxicity or systemic hypotension
			RCTs(24 infants) (44)(13 infants) (45)	•Improved OI: MD = −10 (*p* < 0.05) •Decreased duration of MV: MD = −7.2 days (*p* < 0.001)	•Dose: 1 mg/kg b.i.d, for a mean duration of 4.8 ± 1.1 days (oral)
				•No improvement in oxygenation or other outcomes	•Dose: 2 mg/kg b.i.d, for a mean duration of 5.0 ± 2.6 days (oral) •No hepatotoxicity or systemic hypotension •Delayed absorption of bosentan in critically ill neonates
Glucocorticoids		Anti-inflammatory	Case series (15 infants) (46)	•Increased systolic blood pressure and decreased need for inotropic support (*p* < 0.001) •Improved OI (*p* < 0.001) and PaO_2_/FiO_2_ (*p* < 0.001)	•Hydrocortisone; Dose: 4 mg/kg, followed by 1 mg/kg every 6 hours for 48 h (IV)

*Only statistically significant results are presented. AaDO_2_, alveolar–arterial oxygen difference; ET, endothelin; FiO_2_, fraction of inspired oxygen; iNO, inhaled nitric oxide; IV, intravenous; LV, left ventricle; MD, mean decrease; MR, mean reduction; MV, mechanical ventilation; NO, nitric oxide; OI, oxygenation index; PaO_2_, arterial partial pressure of oxygen; PAP, pulmonary artery pressure; PDE3, phosphodiesterase 3; PDE5, phosphodiesterase 5; PGE1, prostaglandin E1; PGI2, prostacyclin; RCTs, randomized controlled trials; RR, relative risk; RV, right ventricle; SpO_2_, oxygen saturation*.

##### PDE5 inhibitors

PDE5 is a key regulator of newborn pulmonary vascular reactivity, and its inhibition leads to vasodilation. As such, PDE5 inhibitors, mainly sildenafil, are currently studied as PPHN treatment. Sildenafil is a widely recognized agent in the chronic management of adult PAH (48). The most recent meta-analysis on its efficacy and safety included five RCTs, comprising 166 term and late preterm neonates with PPHN (33). It compared sildenafil vs. no treatment, placebo or another pulmonary vasodilator, with or without combined sildenafil. Three studies showed a significant reduction in mortality when comparing sildenafil use to placebo, despite not showing a mortality decrease when compared with the use of iNO, with or without combined sildenafil (33). A steady improvement in oxygenation after the first dose of sildenafil was observed, and no clinically important adverse events were reported (33), although hypotension and pneumothorax were previously described (49). Overall, sildenafil used as treatment of PPHN has potential for reducing mortality and improving oxygenation in these infants. However, to date, the available trials are limited and lack quality of evidence, due to small sample sizes and unreliable methodological features (33).

Currently, and despite the scarce evidence, sildenafil is used as adjuvant in iNO-resistant PPHN or as monotherapy when iNO is not available or contraindicated, as well as in chronic primary treatment of PH associated to conditions such as CDH and bronchopulmonary dysplasia (BPD), in which iNO efficacy is distinctly insufficient (6). In fact, sildenafil has shown to improve pathological features of PPHN in experimental CDH (50) and, given the high mortality and morbidity of this pathology, might be of a valuable potential benefit for these infants. A trial evaluating chronic sildenafil for severe CDH was recently terminated due to the change in clinical recommendations, allowing the use of sildenafil for the treatment of PPHN in CDH neonates (6), incompatible with placebo enrollment (NCT00133679 http://www.clinicaltrials.gov).

Tadalafil is another PDE5 inhibitor approved for adult PAH. Experimentally, it reduces PVR and increases oxygenation and cardiac output in PPHN piglets (51), as described in Table 4. It may be more effective than sildenafil, due to higher selectivity for PDE5 and longer half-life (71). However, no studies in newborns have been performed.

**Table 4:** An overview of potential therapeutic approaches for persistent pulmonary hypertension of the newborn (PPHN), studied in animal models or conceptual.

**Treatment options**	**Mechanism of action**	**Animal model**	**Main results**	**Treatment protocol and relevant observations**
Tadalafil	PDE5 inhibitor	◦Piglets, hypoxia-induced (51)	•Decreased PAP (by 54%) and increased cardiac output (by 88%) (*p* < 0.05) •Increased PaO_2_ (by 48 ± 21%) and decreased AaDO_2_ (by 74 ± 13%) (*p* < 0.01)	•Dose 1 mg/kg (single oral dose) •Higher selectivity for PDE5 and longer half-life
L-citrulline	Exogenous L-arginine (eNOS substrate)	◦Piglets, hypoxia-induced (52)	•Decreased PVR and decreased RV mass (*p* < 0.05) •Increased NO and decreased superoxide (eNOS recoupling) (*p* < 0.05)	•Dose: 1–1.5 g/day for 7 days (oral)
Riociguat	sGC stimulator	◦Lambs, ligated DA (53)	•Decreased PVR (by 60%) and increased PBF (by 2-fold) in lamb models (*p* < 0.01)	•BAY 41-2272: tool-compound for riociguat •Dose: 500 mcg (single infusion in LPA)
Cinaciguat	sGC activator	◦Lambs, ligated DA (54)	•Increased PBF (by 4-fold) and decreased PVR (by 80%) (*p* < 0.01)	•Dose: 150 mcg (single infusion in LPA) •Beneficial effects after oxidative stress
Selexipag	PGI2 receptor agonist	Conceptual	Based on concepts alone	•Oral administration •Promising results in adult PAH, with no apparent safety concerns (55)
Fasudil	ROCK inhibitor	◦Lambs, ligated DA (56)	•Increased PBF (by twofold) and decreased PVR (by 51 ± 11%) (*p* < 0.05)	•Dose: 500 mcg (single infusion in LPA)
		◦Rats, hypoxia-induced (57)	•Decreased PVR (*p* < 0.05)	•Dose: 30 mg/kg (single IV bolus) •Severe adverse effects(systemic hypotension, growth restriction) (58)
Simvastatin	RhoA (and ROCK) inhibitor	◦Rats, hypoxia-induced (59)	•Decreased PVR (either preventive or rescue therapy) (*p* < 0.05) •Decreased RV mass (either preventive or rescue therapy) (*p* < 0.05) •Improved exercise capacity and decreased pulmonary arterial remodeling (if rescue therapy) (*p* < 0.05)	•Dose: 2 mg/kg/day for either 7 (rescue) or 14 (preventive) days (intraperitoneal injection) •No systemic hypotension or apparent toxic effects on skeletal muscle, liver, or brain
Rosiglitazone	PPAR-γ agonist	◦Lambs, ligated DA (60)	•Decreased *in vitro* proliferation of PASMCs (by 51%) (*p* < 0.01)	•Prevents the development of PAH induced by hypoxia (61) or hyperoxia (62) in rats
Sapropterin	Exogenous BH4 (eNOS cofactor)	◦Piglets, hypoxia-induced (63)	•Decreased PVR, decreased PAP, and decreased RV mass (*p* < 0.05) •Increased NO and decreased superoxide (eNOS recoupling) (*p* < 0.05)	•Dose: 20 mg/kg for 1 day, followed by 40 mg/kg/day for 7 days (oral) •Established safety profile in human infants (64)
rhSOD	Exogenous SOD	◦Lambs, ligated DA (65, 66)	•Improved a/A ratio, improved OI and improved PaO_2_ (*p* < 0.05) •Oxygenation improved more rapidly if rhSOD + iNO, compared with either intervention alone (*p* < 0.05)	•Dose: 5 mg/kg (single endotracheal dose) •rhSOD also reduced pulmonary arterial contractility (isolated vessel) and oxidation
			•Decreased PAP (*p* < 0.05) •Decreased PVR if rhSOD + iNO, compared with either intervention alone (*p* < 0.05)	•Dose: 5 mg/kg (single endotracheal dose) •No systemic hypotension
ω-3 LC-PUFAs	Antioxidant Anti-inflammatory	◦Lambs (no PPHN) (67)	•Increased PBF (by 30%) and decreased PVR (by 28%) (*p* < 0.0001)	•Dose (Omegaven®): 2.4 ml (single infusion in LPA)
Ketanserin	5-HT2A antagonist	◦Lambs, ligated DA (68)	•Decreased PAP, decreased PVR (by 26%), and increased PBF (by 27%) (*p* < 0.05)	•Dose: 20 mg (single infusion in LPA) •Systemic hypotension when used for adult PAH (69)
Sarpogrelate	5-HT2A antagonist	Conceptual	Based on concepts alone	•Oral administration •No systemic hypotension (in human adults)
rhVEGF	Exogenous VEGF	◦Lambs, ligated DA (70)	•Decreased PAP (*p* < 0.05) •Increased expression of lung eNOS (*p* < 0.05) and decreased pulmonary artery wall thickness (by 34%) (*p* < 0.01)	•Dose: 15 mcg/day for 14 days (infusion in LPA)

*Only statistically significant results are presented. ω-3 LC-PUFAs, ω-3 long-chain polyunsaturated fatty acids; 5-HT2A, 5-HT receptor 2A; BH4, tetrahydrobiopterin; a/A, arteriolar-to-alveolar oxygen ratio; AaDO_2_, alveolar–arterial oxygen difference; DA, ductus arteriosus; eNOS, endothelial NO synthase; iNO, inhaled nitric oxide; IV, intravenous; LPA, left pulmonary artery; OI, oxygenation index; PaO_2_, arterial partial pressure of oxygen; PAP, pulmonary artery pressure; PBF, pulmonary blood flow; PAH, pulmonary arterial hypertension; PPAR-γ, peroxisome proliferator-activated receptor-γ; PVR, pulmonary vascular resistance; PDE5, phosphodiesterase 5; PGI2, prostacyclin; RhoA, Ras homologue family member A; ROCK, Rho-kinase; rhSOD, recombinant superoxide dismutase; rhVEGF, recombinant vascular endothelial growth factor; ROCK, Rho-kinase; RV, right ventricle; sGC, soluble guanylyl cyclase*.

Further RCTs investigating PDE5 inhibitors should be performed to increase the quality of evidence, as well as to study its long-term effects in infants. A multicenter RCT to evaluate efficacy, safety, and long-term developmental progress after use of intravenous sildenafil in the treatment of neonates with PPHN is estimated to be completed later this year (NCT01720524 http://www.clinicaltrials.gov).

##### L-Citrulline and sGC activators/stimulators

Two other substances with the ability to interfere in NO/cGMP cascade ameliorate PPHN in animal models, although both never tested in human newborns: l-citrulline and sGC activators/stimulators.

l-Citrulline is converted to l-arginine, the substrate of eNOS, to produce NO (1). l-Citrulline is transported into PAECs by a sodium-coupled neutral amino acid transporter (SNAT1), which responds to hypoxia, modulating NO production (72). It has been demonstrated that impaired l-citrulline–l-arginine-NO pathway is involved in the pathogenesis of PPHN (73). l-Citrulline promotes eNOS recoupling in PPHN, improving NO production, and ameliorates hypoxia-induced PPHN when used as rescue therapy in newborn piglets (52). These findings suggest that, by increasing NO production, supplemental l-citrulline or modulators of its transporter SNAT1 may be promising alternate therapies for newborns with PPHN. Although oral l-citrulline has shown to reduce PVR and improve functional capacity in adult PAH (74), no trials regarding its use in newborns have been performed.

sGC is the target enzyme of NO in PASMCs. However, tolerance or resistance to NO may limit sGC activity and cGMP production in PASMCs, hence vasodilation (47). In lamb models of PPHN, sGC activity is impaired (75), warranting interest on sGC direct stimulators/activators as potential therapies, allowing a bypass to NO. Riociguat is an sGC stimulator, already approved for the treatment of adult PAH, although its effects were never studied in children or infants (53, 76). On its turn, cinaciguat is an sGC activator, leading to sustained pulmonary vasodilation in the lamb model of PPHN (54). iNO use is optimal in the absence of oxidative stress since it further contributes to the generation of ROS and uncoupling of eNOS. Contrariwise, cinaciguat increases cGMP production in PASMCs from PPHN lambs, leading to vasodilation, even in the presence of oxidative stress due to hyperoxia (75). These findings suggest that sGC stimulators/activators may be potentially used as alternative or adjuvant therapy for infants with iNO-resistant PPHN, while cinaciguat may provide a treatment option for PPHN aggravated by exposure to hyperoxia. However, both riociguat and cinaciguat warrant further investigation on efficacy and long-term effects before its clinical use in infants.

### Arachidonic Acid–Prostacyclin–cAMP Pathway

#### Pathway

**Figure 3:**
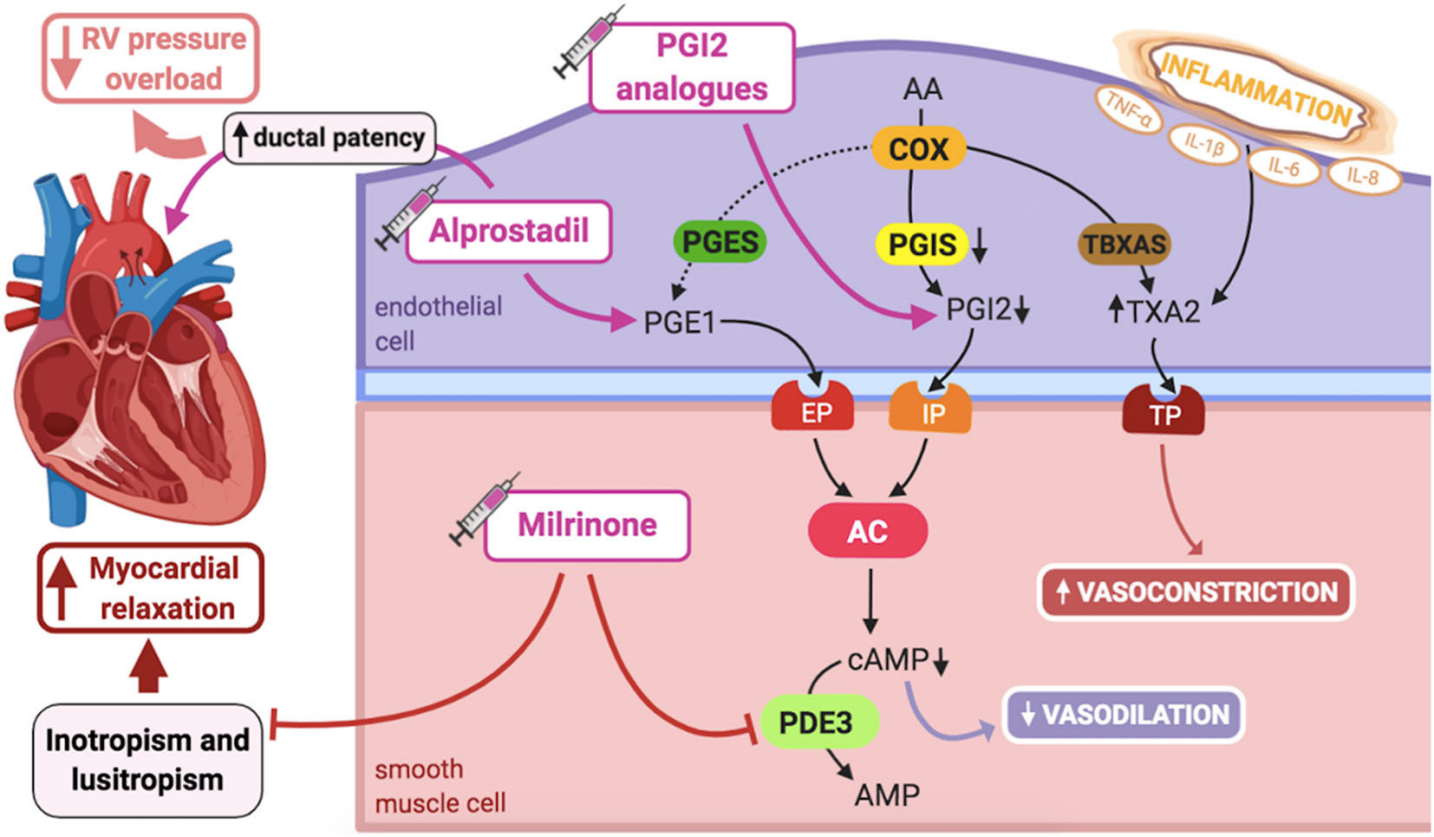
Pathogenic mechanisms of persistent pulmonary hypertension of the newborn (PPHN) and its current and potential target therapies: Arachidonic acid-prostacyclin-cAMP pathway. AA, arachidonic acid; AC, adenylyl cyclase; AMP, adenosine monophosphate; cAMP, cyclic adenosine monophosphate; COX, cyclooxygenase; EP, PGE1-receptor; IL-1β, IL-6, IL-8, interleukins-1β, 6 and 8; IP, PGI2-receptor; PDE3, phosphodiesterase-3; PGE1, prostaglandin E1; PGES, PGE1 synthase; PGI2, prostacyclin; PGIS, PGI2 synthase; RV, right ventricle; TBXAS, TXA2 synthase; TNF-α: tumor necrosis factor α; TP, TXA2-receptor; TXA2, thromboxane A2. Target therapies are marked with a syringe icon and are colored based on the type of evidence supporting its use on PPHN- Purple: Evidence on its was obtained by adequately powered RCTs/meta-analysis; Pink: Evidence limited to observational studies or small and underpowered RCTs and/or inconsistent results in human newborns; Blue: Beneficial effects only demonstrated in experimental models of PPHN. This figure was created with BioRender.com.

The arachidonic acid–prostacyclin–cAMP pathway plays an important role in vascular pulmonary tonus (Figure 3). Prostaglandins activate adenylyl cyclase, increasing intracellular cAMP in PASMCs, resulting in vasodilation (1). Phosphodiesterase-3 (PDE3) breaks down cAMP, thus limiting the duration of cAMP-induced vasodilation. Prostacyclin (PGI2) is the most potent vasodilator prostaglandin (1).

PGI2 partly mediates pulmonary vasodilation at birth, in a complementary fashion to the NO-cGMP system (1). However, unlike NO-cGMP, it does not seem to markedly respond to oxygenation, but mainly to the increased PBF. Although this pathway does not seem crucial for maintaining PVR *in utero*, PGI2 participates in its decline at birth (25).

In lamb models of PPHN, pulmonary prostacyclin synthase (PGIS) and PGI2-receptor expression in the lung are decreased, leading to reduced vasodilation in response to PGI2 analogs, although adenylate cyclase expression is preserved. However, pretreatment with milrinone, a PDE3 inhibitor, by increasing cAMP levels, is able to fully restore lung vasodilation in response to prostanoids in these animals (77). Accordingly, dysregulation of PDE3 expression and activity can interfere with normal cAMP signaling in PASMCs, although levels of PDE3 do not seem to be altered in PPHN fetal lambs (78).

#### Potential Targeted Therapies

##### Prostaglandin analogs

The effects of interfering with prostacyclin–cAMP pathway can be complementary to iNO, since they stimulate different cyclic nucleotides: cAMP and cGMP, respectively. Two classes of prostaglandin analogs, PGI2 and prostaglandin E1 (PGE1), have been studied in the context of PPHN.

PGI2 analogs are currently the backbone of pulmonary vasodilator therapy for PAH in adults and children (47). Small studies, mostly observational, as well as experimental findings, have been suggesting PGI2 analogs as valuable candidates for PPHN treatment (34–39, 79), as described in Table 3. Particularly, some have shown to improve oxygenation in iNO-resistant infants, who might have impaired cGMP-mediated pulmonary vasodilation, hence could specially benefit from PGI2 analogs, acting through cAMP signaling (37–39).

A meta-analysis was recently published, aiming to determine the efficacy and safety of PGI2 analogs (iloprost, treprostinil, and beraprost) in decreasing mortality and the need for ECMO among neonates with PPHN. However, it did not identify eligible neonatal trials, concluding that there is not enough evidence supporting their safety and efficacy as pulmonary vasodilators in this population. As such, adequately powered, multicenter RCTs are needed to address this question, as well as to study their effects on short-term outcomes, such as mortality, and long-term outcomes, either neurodevelopmental or pulmonary (80).

An RCT studying intravenous treprostinil (Remodulin®) as adjuvant to iNO in the treatment of term or near-term infants with PPHN is now recruiting and estimated to be completed in 2022 (NCT02261883 http://www.clinicaltrials.gov).

PGE1 analog alprostadil is widely used to maintain patency of *ductus arteriosus* in newborns with cyanotic congenital heart disease. Besides the pulmonary vasodilator effects of PGE1, ductal patency may offer an advantage for these neonates by reducing RV pressure overload (47). A retrospective study has shown that PGE1 treatment associates with significantly shortened length of stay when used in infants with PPHN without associated congenital heart disease, although with no significant decrease in mortality (40). There is also a small pilot study demonstrating that inhaled PGE1 is a safe and selective pulmonary vasodilator in infants with PPHN (81).

Thus, although prostaglandin analogs are recognized pulmonary vasodilators, the literature is currently insufficient on supporting its role as a therapeutic weapon for PPHN. Besides, the associated systemic hypotension observed in some infants treated with PGI2 analogs may limit their use in neonates with PPHN.

Selexipag, an orally administered prodrug of a PGI2 receptor agonist, has been recently studied, and showing encouraging results, for use in the treatment of PAH in adults (55, 82). However, no animal (or human) studies investigating selexipag use in PPHN have been found, although theoretically it may have potential for the treatment of this condition.

##### PDE3 inhibitors

Milrinone is a PDE3 inhibitor, leading to increased cAMP levels in PASMCs and increased vasodilation, besides its inotropic and lusitropic effects, hence contributing to myocardial relaxation (47).

In lamb models with PPHN, milrinone, either intravenous or aerosolized, enhanced vasodilation induced by PGI2 and was not associated with a significant decrease in systemic blood pressure (83, 84). Expression and activity of adenylate cyclase and PDE3 are not decreased in PPHN lambs, suggesting that milrinone target might be unaltered in this condition, most likely allowing an adequate response to its action (77). Thus, animal studies support that combination of milrinone and PGI2 may be a prospective treatment option for PPHN.

Retrospective series of 24 case reports regarding milrinone's effectiveness as a therapy for PPHN showed consistent improvement in oxygenation and no events of systemic hypotension. However, a few cases developed intracerebral hemorrhage (41, 85), raising some safety concerns on the use of milrinone in this population.

Milrinone's use was also studied in 11 neonates with iNO-resistant PPHN, allowing a significant improvement in oxygenation and cardiac output, as well as decreased PAP, with no reported systemic hypotension or intraventricular hemorrhage. However, this study had no control group, so milrinone should not yet be considered as an alternative to iNO, although it seems to improve the clinical outcome in infants resistant to its treatment (42). The same adjuvant effect was also observed in a series of preterm infants (86).

Exposure to iNO in lamb models enhances PDE3 expression and activity (78), which demonstrates the interrelated nature of cGMP and cAMP pathways in PPHN and might partially explain the increased efficacy of milrinone in iNO-resistant PPHN (42).

Finally, in infants with CDH, a known etiology of iNO-resistant PH, improved oxygenation and RV diastolic function after milrinone infusion was recently reported, after previous treatment with iNO, sildenafil, or both (87). In fact, milrinone is commonly used *off label* in the management of CDH infants, although no RCTs have yet confirmed its benefit (47).

These findings suggest that milrinone is an effective therapeutic option in infants with PPHN, particularly if iNO-resistant (as in the case of CDH) or in the presence of cardiac dysfunction, considering its adjuvant effects on myocardium. However, RCTs to evaluate its efficacy and long-term sequelae when used in neonates are warranted before advocating its clinical use. An RCT aiming to establish milrinone's safety and efficacy in improving oxygenation in CDH infants is currently recruiting and estimated to be completed early in 2021 (NCT02951130 http://www.clinicaltrials.gov).

### Endothelin-1-ET_A_/ET_B_ Receptors

#### Pathway

In addition to the impairment of vasodilator mechanisms already discussed, increased vasoconstrictor pathways have also been implicated in the dysregulation of perinatal pulmonary vascular tone involved in the pathogenesis of PPHN.

**Figure 4:**
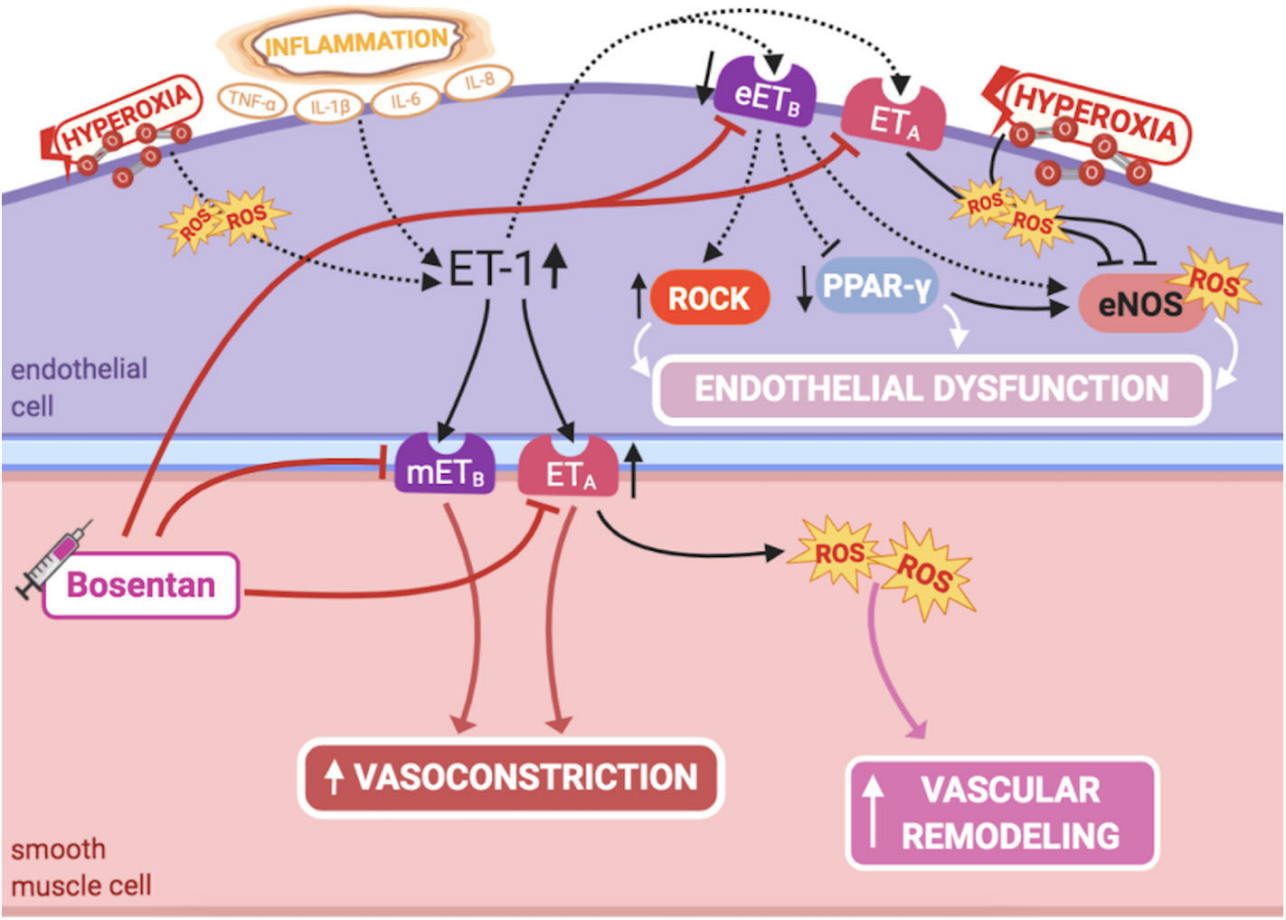
Pathogenic mechanisms of persistent pulmonary hypertension of the newborn (PPHN) and its current and potential target therapies: endothelin-1-ETA/ETB receptors. eETB, endothelial relaxant endothelin receptor B; eNOS, nitric oxide synthase; ET-1, endothelin-1; ETA, endothelin receptor A; IL-1β, IL-6, IL-8, interleukins-1β, 6 and 8; mETB, smooth muscle contractile endothelin receptor B; PPAR-γ, peroxisome proliferator-activated receptor-γ; ROCK, Rho-kinase; ROS, reactive oxygen species; TNF-α, tumor necrosis factor α. Target therapies are marked with a syringe icon and are colored based on the type of evidence supporting its use on PPHN—purple, evidence on its was obtained by adequately powered RCTs/meta-analysis; pink, evidence limited to observational studies or small and underpowered RCTs and/or inconsistent results in human newborns; blue, beneficial effects only demonstrated in experimental models of PPHN. This figure was created with BioRender.com.

ET-1 is a potent vasoconstrictor, produced by PAECs, and comitogen for PASMC hyperplasia, playing a major role in fetal pulmonary vasoregulation (1) (Figure 4). Although ET-1 plays its predominant effect as a pulmonary vasoconstrictor in the normal fetus, it can cause dual pulmonary vascular effects, due to the activation of two different classes of ET receptors: ET_A_ receptor, located on PASMCS, leads to marked vasoconstriction, while ET_B_ receptor mediates the ET-1 vasodilator response, by increasing NO release in PAECs (1). Hence, selective blockade of the ET_A_ receptor causes fetal pulmonary vasodilation (88).

NO-cGMP and ET-1 signaling pathways have complex interactions, regulating each other through autocrine feedback loops (1). NO decreases ET-1 production in PAECs, while ET-1 increases superoxide production through ET_A_ receptor (1), impairing NO production by eNOS (89, 90). Furthermore, the mitogenic effect on PASMCs through ET_A_ receptors is mediated by this increased production of superoxide, which in turn stimulates activation of mitogen-activated protein(MAP) kinases (91). This has shown to be prevented by antioxidant treatment or NADPH oxidase inhibition (91), as well as by NO (92). On the other hand, hyperoxia potentiates ET-1 signaling and diminishes eNOS expression (93). Further understanding of these interactions may lead to novel strategies to treat PPHN.

Increased plasma levels of ET-1 are observed in infants with PPHN (94), inclusively those with CDH (95), and are thought to be a marker of disease severity and poor prognosis (94, 95). Supporting these findings, lung ET-1 levels are markedly increased in lamb models of PPHN, and ET_B_ protein is decreased in their PAECs (96). This altered balance, with increased ET_A_-mediated vasoconstriction and decreased ET_B_-mediated vasodilation, favors increased vascular tone and PASMC proliferation (88, 97). Therefore, upregulation of ET-1 signaling pathway, mainly through ET_A_ stimulation, contributes to PPHN pathogenesis.

#### Potential Targeted Therapies

##### Endothelin receptor antagonists

Modulation of ET-1 pathway, essentially with ET receptor antagonists, could be of interest for PPHN therapy, as an alternative or as adjuvant to iNO.

As such, selective chronic inhibition of ET_A_ receptor has shown to attenuate the severity of PPHN, improving vasodilation at birth (restoring PVR decline) and decreasing pulmonary artery wall thickening and RV hypertrophy in the lamb model of PPHN (97). However, it is becoming increasingly recognized that ET_B_ activation, despite its already discussed vasodilator role, may also be of interest in the pathogenesis of PPHN. As will be later discussed in the respective sections, ET-1 activation of the ET_B_ receptor in PPHN lamb PAECs leads to Rho-kinase (ROCK) activation (98), as well as decreased peroxisome proliferator-activated receptor-γ (PPAR-γ) expression and activity (99), both involved in vasoconstriction and abnormal vascular development. Accordingly, ET_B_ activation is also suggested to be involved in the pathogenesis of PPHN with impaired vascular growth. This implies that combined ET_A_/ET_B_ receptor blockade may be more advantageous than selective ET_A_ receptor inhibition in the context of PPHN.

Bosentan is a non-selective ET-1 receptor antagonist, commonly used for adult PAH (47). Bosentan is only available for oral administration (47), possibly limiting its use in the acute management of newborns with PPHN (45). In term and near-term infants, evidence of its efficacy and safety is limited mostly to retrospective observational studies and two small RCTs (100).

Maneenil et al. have recently reported a case series of 40 infants treated with bosentan, either alone or as adjuvant to iNO, when its response was not satisfactory (43). There was a significant improvement in oxygenation, with no significant change in blood pressure or other apparent adverse events (43). However, this was a small, methodologically poor retrospective study, with neither control group nor evaluation of long-term outcomes (43).

Mohamed et al., in a prospective RCT, showed improved short-term outcomes, such as oxygenation and need for mechanical ventilation, in infants who received bosentan, compared to placebo, in a setting in which iNO and ECMO were not available. Its benefit on long-term outcomes, such as neurodevelopment sequelae, was less clear, and no difference in mortality was found (44). Steinhorn et al. failed to show an additive effect of bosentan to iNO, not reducing the duration of iNO therapy and mechanical ventilation or the need for ECMO (45). Both trials reported no major adverse effects of bosentan. However, due to their small sample sizes, these studies might have overlooked some serious adverse events and do not allow to ascertain the safety of its use in this population, as concluded in a meta-analysis published in 2016 (100). This is particularly relevant since bosentan's use in adults may result, among others, in hepatotoxicity (47).

Bosentan may be of particular interest in the chronic management of infants with iNO-resistant PH (47), associated with BPD or CDH, or in resource-poor sites where iNO is not available, as described earlier in observational studies (43).

Further RCTs have to be conducted to assure bosentan's efficacy and safety in the neonatal population, although no trials are currently on course. Besides, other non-selective antagonists, such as macitentan, or even selective ET-A blockers, such as ambrisentan, have been approved for adult PAH (47). However, none has been studied for the treatment of PPHN; nonetheless, it could potentially be of interest in this context.

### RhoA/ROCK Signaling Pathway

#### Pathway

**Figure 5:**
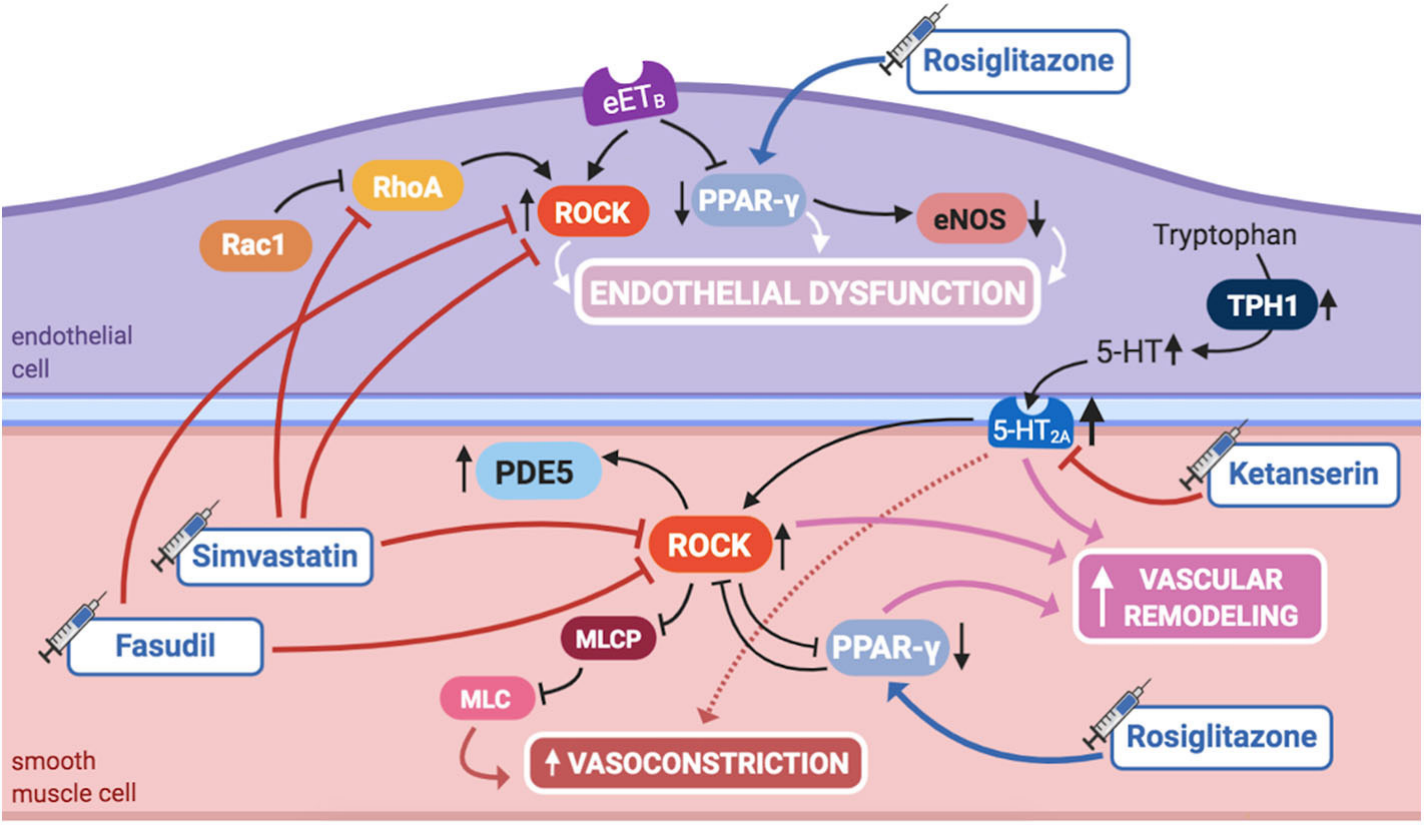
Pathogenic mechanisms of persistent pulmonary hypertension of the newborn (PPHN) and its current and potential target therapies: RhoA/ROCK signaling, PPAR-γ, and 5-HT signaling pathways. 5,HT, serotonin; 5-HT2A, 5-HT receptor 2A; eETB, endothelial relaxant endothelin receptor B; eNOS, nitric oxide synthase; ET-1, endothelin-1; ETA, endothelin receptor A; IL-1β, IL-6, IL-8, interleukins-1β, 6, and 8; mETB, smooth muscle contractile endothelin receptor B; MLC, myosin light chain; MLCP: myosin light chain phosphatase; PDE5, phosphodiesterase-5; PPAR- γ, peroxisome proliferator-activated receptor γ; Rac1, Ras-related C3 botulinum toxin substrate 1; RhoA, Ras homolog family member A; ROCK, Rho-kinase; TNF-α: tumor necrosis factor α; TPH1, tryptophan hydrolase 1. Target therapies are marked with a syringe icon and are colored based on the type of evidence supporting its use on PPHN—purple, evidence on its was obtained by adequately powered RCTs/meta-analysis; pink, evidence limited to observational studies or small and underpowered RCTs and/or inconsistent results in human newborns; blue, beneficial effects only demonstrated in experimental models of PPHN. This figure was created with BioRender.com.

ROCK plays a significant role in controlling the pulmonary vascular tone and structure (Figure 5). When activated, it inhibits myosin light-chain phosphatase, blocking PASMCs' relaxation, hence promoting vasoconstriction. RhoA is a GTPase that increases ROCK activity, while Rac1, also a GTPase, decreases RhoA activity, therefore ultimately contributing to diminished ROCK activity.

ROCK inhibition has shown to prevent the development of PAH in adult rats (101). More recently, it has been demonstrated that ROCK's activity is increased in several animal models of PPHN, and its inhibition during fetal life enhances pulmonary vasodilation, ameliorating PPHN (56, 57), which suggests that ROCK may also play a role in maintaining high PVR *in utero*.

A sustained increase in RhoA activity, as well as a reduction in Rac1 activity, was found in PAECs from piglets with hypoxia-induced PPHN (102). Inhibition of RhoA, as well as activation of Rac1, restored hypertensive PAECs' phenotype to normal, infatuating the eventual role of RhoA/ROCK pathway modulation as an alternative therapy for PPHN (102).

RhoA/ROCK pathway inhibitory agents also reduce vasoconstriction when NO production is blocked in the perinatal lung, opening doors for possible interactions between these two signaling pathways (56) and suggesting ROCK inhibitors as a potential treatment.

Moreover, RhoA/ROCK pathway is also one of the effectors of serotonin (5-HT) signaling, another aspect of PPHN pathophysiology. This was long known in adult PASMCs and more recently suggested in experimental PPHN. 5-HT causes potent vasoconstriction in the ovine fetal pulmonary circulation through activation of the 5-HT_2A_ receptor, which, at least in part through ROCK signaling, contributes to sustained abnormalities of pulmonary vascular tone, reactivity, or structure, leading to PPHN (103).

Further evidence suggests an additional interaction of RhoA/ROCK pathway, with PPAR-γ (60), another molecule involved in PPHN pathophysiology, as discussed in this review. ROCK inhibition in PPHN fetal lambs with decreased PPAR-γ activity has shown to increase it, restoring it to normal, consequently reinstating the natural growth of PASMCs (60).

Finally, PPHN, if untreated, culminates in RV failure and death. In a model of rat pups exposed to neonatal hypoxia, RhoA as well as Rho-kinase activity were both increased in the right, but not left, cardiac ventricle, most likely playing a role on RV hypertrophy and systolic dysfunction. This might be explained, in part, by upregulation of PDE5 activity downstream of ROCK activation. Rho-kinase inhibition in this model decreased PDE5 activity, normalized PVR, and caused regression of RV hypertrophy and pulmonary arterial wall remodeling, although it was not able to ameliorate systolic function, contrary to sildenafil (58).

#### Potential Targeted Therapies

##### Rho-kinase inhibitors

Accordingly, the activity of ROCK or its modulating molecules, such as RhoA, may present as potential future targets for PPHN treatment. As already stated, many experimental uses of Rho-kinase inhibitors, such as Y-27632 or fasudil, have been tried, either in neonatal rats, as in ovine fetal PPHN models. Results have been quite promising on reducing the functional and structural lung abnormalities commonly associated with PPHN, not only when used in combination to therapies targeting interacting pathways, such as NO/GMP or ET-1, but also when used independently (56–58, 98). Besides, fasudil attenuates 5-HT-mediated vasoconstriction in the ovine fetus model (103). However, in neonatal rats, ROCK inhibitors produce severe adverse effects, including systemic hypotension and growth restriction (57, 58), which may limit its use as a pulmonary vasodilator in neonates with PPHN.

Fasudil is already approved for the treatment of adult PAH, significantly improving pulmonary hemodynamics without significant toxicity (104, 105). However, there are no studies investigating its effects in infants with PPHN. As such, despite having experimental potential, further research, such as a large double-blinded RCT, is required to investigate the effect of fasudil vs. placebo or iNO in PPHN, as well as to access its potential adverse effects in the newborn population with its distinctive physiology.

Besides the classical ROCK inhibitors, simvastatin was also tested for this purpose in a recent study in neonatal rats, since it decreases RhoA's activity. In this model, simvastatin, used as either preventive or rescue therapy, has shown to decrease RhoA/ROCK signaling in hypoxia-induced PPHN, decreasing PVR, RV hypertrophy, and pulmonary arterial remodeling (59). In addition, it significantly improved exercise capacity and did not cause apparent toxic effects, such as hypotension or growth restriction, nor alterations in the total cholesterol and low-density lipoprotein (LDL) serum levels, which would be deleterious for brain development in newborns. In fact, simvastatin restored normal growth in chronic hypoxia-exposed rats (59). Although there were no reported adverse effects of simvastatin when investigated in children with PAH (106), further study is needed specifically in the newborn population in order to sustain its safety, as well as efficacy, as a potential treatment for PPHN.

### Peroxisome Proliferator-Activated Receptor-γ

#### Pathway

PPAR-γ is a transcription factor that regulates lung and alveolar development (107), also regulating pulmonary vascular tone and decreasing PASMCs' proliferation (108), thus inhibiting vascular remodeling (Figure 5). In a mouse model, knockout of PPAR-γ resulted in spontaneously developed PAH and RV hypertrophy (108), whereas its activation by rosiglitazone, a widely acknowledged PPAR-γ agonist used in the treatment of diabetes, has prevented the development of PAH induced by hypoxia (61) or hyperoxia (62).

Moreover, some PPHN patients show variants in the genes of corticotrophin-releasing hormone receptor 1 and its binding protein (CRHR1 and CRHBP), determining decreased expression of PPAR-γ, which might further contribute to its role in the pathogenesis of PPHN (109).

PPAR-γ regulates NO production by modulating eNOS expression and activity in fetal PAECs, and its agonists increase its activity *in vitro*, hence endogenous NO production in PPHN lamb models (99). Additionally, PAECs in PPHN lamb models are characterized by impaired ability to form vascular structures, at least in part by decreased PPAR-γ expression and activity. ET-1 administration reproduces these effects, impairing PAECs' function, which is prevented by PPAR-γ agonist treatment (99). These findings imply that ET-1 decreases PPAR-γ signaling, contributing to PAECs' dysfunction and impaired angiogenesis in PPHN, suggesting that ET-1/PPAR-γ interactions regulate PPAR-γ-dependent eNOS activity, NO production, and vascular formation (99).

Furthermore, it has been established that inhibition of PPAR-γ expression in PAECs of PPHN lamb models is secondary to ET-1 activation of the ET_B_ receptor (99), similarly to the already discussed activation of ROCK (98). This further supports that combined ET_A_/ET_B_ receptor blockade may be more beneficial than selective ET_A_ receptor blockade in this context.

#### Potential Targeted Therapies

##### PPAR-γ agonists

Therapies that restore PPAR-γ signaling, either by using direct PPAR-γ agonists or by inhibiting ET-1 activity, may have a potential role in the treatment of PPHN, while a combination of PPAR-γ agonists and bosentan may be useful.

Besides, interactions between ROCK and PPAR-γ have been found in PPHN lambs, since ROCK inhibition restores PPAR-γ activity in these models (60). Additionally, PPAR-γ agonists seem to produce vasodilation through inhibition of ROCK, implying that there might be several points of intersection between these two axes (110). These findings suggest that combined therapy with ROCK inhibitors and PPAR-γ agonists may also be more beneficial in the treatment of PPHN than either agents used alone.

Although all these experimental studies demonstrate a potential role for PPAR-γ agonists, such as rosiglitazone and 15D-prostaglandin-J2, in the treatment of PPHN, no studies involving human infants have yet been conducted. However, experience of rosiglitazone's use in adult patients raises cardiovascular concerns, which may limit its translation to clinical use in the newborn population.

### Hyperoxia and Reactive Oxygen Species

#### Pathway

PPHN is associated with severe hypoxemia, so ventilation with high oxygen concentrations (up to 100%) is common in these neonates (6). However, hyperoxia exacerbates oxidative stress in the affected vasculature, leading to increased production of ROS. Newborns are particularly at increased risk for amplified oxidative stress, since extrauterine alveolar oxygen tension is five times higher than that *in utero*. As such, the fetal lung, during late gestation, goes through adaptive increases in cellular antioxidant defenses, mostly superoxide dismutase-2 (SOD2) (111).

**Figure 6:**
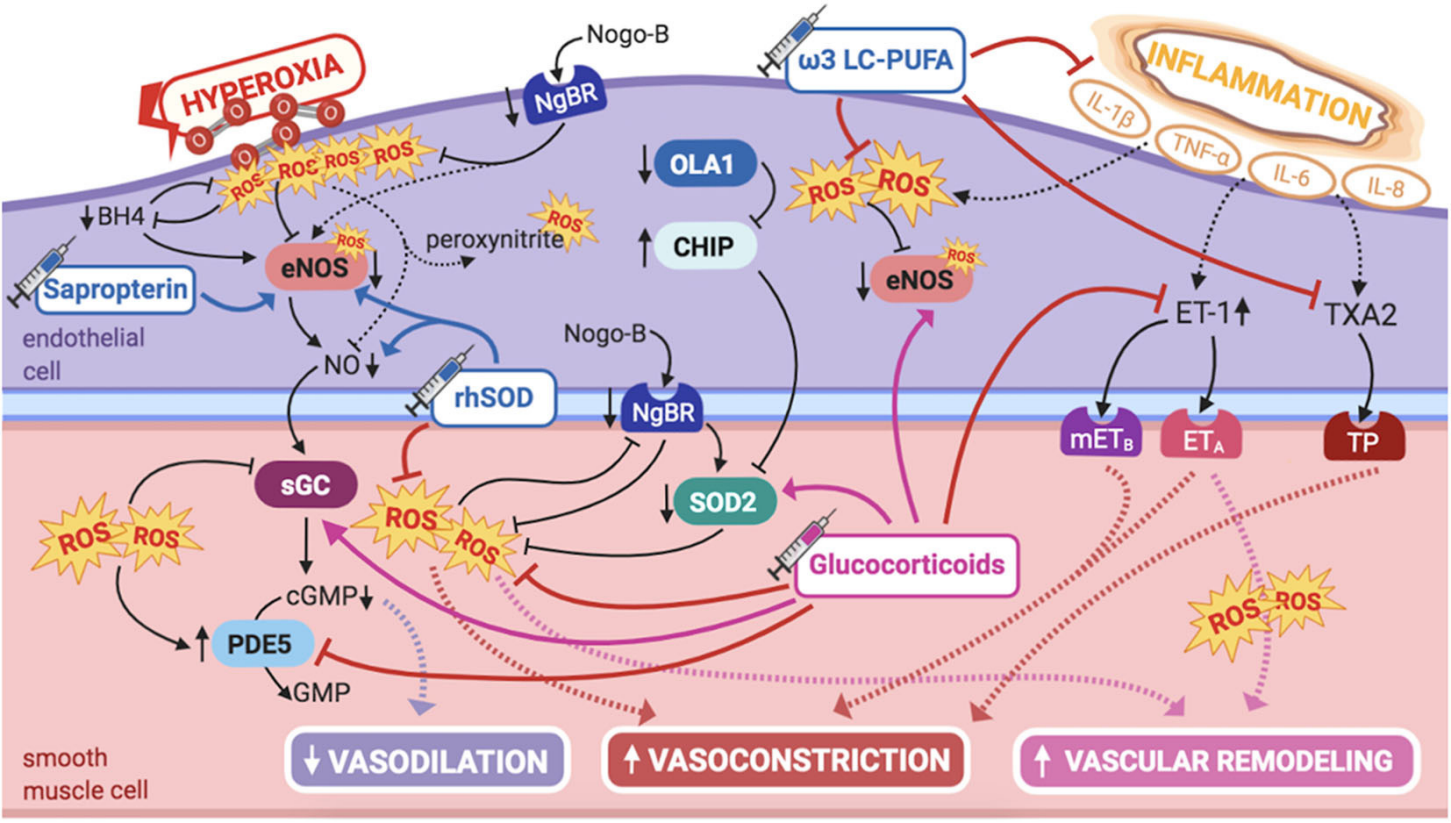
Pathogenic mechanisms of persistent pulmonary hypertension of the newborn (PPHN) and its current and potential target therapies: perinatal inflammation and hyperoxia and reactive oxygen species (ROS). ω-3 LC-PUFAs, ω-3 long-chain polyunsaturated fatty acids; 5-HT, serotonin; 5-HT2A, 5-HT receptor 2A; BH4, tetrahydrobiopterin; cGMP, cyclic guanylyl monophosphate; CHIP, C terminus of Hsc70-interacting protein; eNOS, nitric oxide synthase; ET-1, endothelin-1; ETA, endothelin receptor A; GMP, guanylyl monophosphate; IL-1β, IL-6, IL-8, interleukins-1β, 6, and 8; mETB, smooth muscle contractile endothelin receptor B; NgBR, Nogo-B receptor; NO, nitric oxide; OLA1, Obg-like ATPase 1; PDE5, phosphodiesterase-5; rhSOD, recombinant superoxide-dismutase; ROS, reactive oxygen species; sGC, soluble guanylyl cyclase; SOD2, superoxide-dismutase 2; TNF-α: tumor necrosis factor α; TP, TXA2-receptor; TXA2, thromboxane A2. Target therapies are marked with a syringe icon and are colored based on the type of evidence supporting its use on PPHN—purple, evidence on its was obtained by adequately powered RCTs/meta-analysis; pink, evidence limited to observational studies or small and underpowered RCTs and/or inconsistent results in human newborns; blue, beneficial effects only demonstrated in experimental models of PPHN. This figure was created with BioRender.com.

ROS, such as superoxide, are increased in PPHN lamb (112) and rat models (113), so that many signaling pathways involved in PPHN pathogenesis may act, at least in part, by increasing ROS' levels in multiple cellular compartments of the pulmonary vasculature, ultimately leading to vasoconstriction and PASMCs proliferation (Figure 6).

Besides the direct effects on pulmonary vasculature, exposure to hyperoxia in premature rats results in mitochondrial dysregulation that persists into adulthood with eventual RV dysfunction, suggesting a direct deleterious effect on the RV (114). This effect is most likely cumulative with the direct contribution from the coexisting PPHN, since mitochondrial dysfunction and metabolic gene reprogramming in the RV have previously been identified in adults with PAH (115).

During the transition to extrauterine life, increased PaO_2_ contributes to increased endogenous NO release, hence vasodilation and increased PBF. However, exposure to hyperoxia in newborn lambs results in pulmonary vasoconstriction, hence contributing to the pathogenesis of PPHN, reducing the ability of PAECs to respond to NO (endogenous and exogenous) by several mechanisms (116). Superoxide interacts with and inactivates NO, producing peroxynitrite, not only causing vasoconstriction but also surfactant inactivation (116). Moreover, ROS interfere with NO/GMP pathway enzymes, decreasing eNOS and sGC activities, as well as significantly increasing PDE5 activity (89). Altogether, this results in decreased cGMP levels, further potentiating pulmonary vasoconstriction. However, sildenafil has been demonstrated to restore vascular cGMP signaling and to reduce RV hypertrophy in a hyperoxia-induced PPHN murine model (89).

Besides the interference with NO/cGMP system, hyperoxia potentiates PAF receptor-mediated effects in newborn ovine PASMCs. Exposure to hyperoxia disrupts the normal crosstalk between PAFR-mediated and cAMP/PKA-mediated axes, resulting in increased PASMCs' proliferation. This culminates in vascular hypertrophy and dysfunction, critical for the onset and maintenance of PPHN (117).

Therefore, both extremes of oxygen content in the neonatal lung should be avoided: hypoxia is, of course, a well-acknowledged pathogenic factor in PPHN; however, in recent years, hyperoxia has also been extensively studied and might be just as harmful as hypoxia for the onset and perpetuation of pulmonary vascular dysfunction characteristic of PPHN.

#### Potential Targeted Therapies

##### Tetrahydrobiopterin

eNOS is uncoupled, hence inactivated, by ROS in the presence of oxidative stress, which points for a potential therapeutic approach. Low tyrosine and high phenylalanine levels observed in infants with PPHN could be explained by decreased BH4 levels, hence impaired phenylalanine hydroxylase activity (118, 119). BH4 is a reducing substance, thus intracellular antioxidant, depleted by oxidative stress. BH4 acts as a cofactor for eNOS, playing an essential role in modulating pulmonary vascular tone and being critical in the pathogenesis of adult PAH (119). Oxidation/depletion of BH4 by oxidative stress uncouples eNOS activity, explaining, at least in part, eNOS dysfunction due to oxidative stress, shifting its normal NO production into superoxide formation, and contributing to mitochondrial dysfunction, further amplifying oxidative stress in the pulmonary vasculature (28, 120).

Therefore, these infants may benefit from supplementation with BH4, a potential therapeutic target for PPHN. Sapropterin, an oral BH4 compound, has shown to have beneficial effects on ameliorating hypoxia-induced PPHN in newborn piglets, at least in part by promoting eNOS recoupling in the pulmonary vasculature (63). Moreover, the safety profile of sapropterin's long-term use has been established in human infants with metabolic disorders (64). Besides investigating the potential use of BH4 in newborn infants with hypoxia-induced PPHN, it would also be interesting to study its effects when exposure to hyperoxia, and its consequent increased ROS formation, is involved in pulmonary vascular damage.

An antioxidant therapeutic approach, scavenging superoxide, may, of course, be of beneficial use in this context, reducing oxidative stress and potentially increasing the availability of both endogenous and iNO, limiting lung injury, and improving response to therapy. Besides BH4, some other antioxidants such as *N*-acetylcysteine, apocynin, and ascorbate have shown to decrease intracellular ROS and improve NO bioavailability, but their efficacy has not yet been demonstrated in human infants (121).

##### Recombinant superoxide dismutase

SOD2 expression is decreased in the pulmonary vasculature of PPHN human infants, as well as in the lamb model (122), contributing to endothelial dysfunction and impaired NO-dependent vasodilation (122). This is not surprising, since SOD2 is the first line of defense against ROS, maintaining the balance of NO vs. superoxide during transition to extrauterine life (111). Accordingly, these observations had implications in the research for alternative therapies.

More than a decade ago, Lakshminrusimha et al. demonstrated that intratracheal recombinant superoxide dismutase (rhSOD) could, independently, reduce vasoconstriction and oxidation in newborn lambs with PPHN, besides increasing iNO efficacy, when combined (65). This enhanced effect of rhSOD + iNO, observed for the first time by Steinhorn et al. (66), is probably explained by the fact that iNO therapy further potentiates oxidative injury, which might be ameliorated when rhSOD is used as adjuvant. Inhaled rhSOD restores eNOS expression and markedly decreases pulmonary vascular tone in this model (123).

These findings indicate that rhSOD, by decreasing oxidative stress and restoring eNOS coupling, could be a potential therapy for PPHN, at least as adjuvant to iNO. However, evidence is limited to the lamb model of PPHN, as its use was never studied in humans. To date, no clinical trial investigating rhSOD has been found, although the initial findings on rhSOD experimental efficacy date almost 20 years.

On the same line of thought, proteostatic downregulation of SOD2 is a potential mechanism contributing to PPHN, as shown in infants and lambs (124). This downregulation seems to be related to ubiquitin–proteasome pathways, resulting from decreased OLA1 expression and subsequent enhanced C terminus of Hsc70-interacting protein (CHIP)-mediated degradation of SOD2 and Hsp70, the chaperone that promotes its folding and transport (124). In the lamb model, these abnormalities occur early in the disease process, impairing ROS metabolism by the mitochondria, hence creating an oxidative stress environment that leads to PAECs' apoptosis and PASMCs' proliferation (124).

While the experimental therapeutic benefit of exogenous SOD2 in PPHN is widely recognized, these recent findings allow perspectives for an interesting alternative, focused on increasing endogenous SOD2 activity. The observations that increasing OLA1 expression or reducing CHIP expression restore SOD2 function (124) suggest that its inactivation is possibly reversible and that OLA1 and CHIP are potential therapeutic targets for PPHN, to be explored in future studies.

### Perinatal Inflammation

#### Pathway

Perinatal sepsis and MAS are two of the most recognized etiologies of PPHN, both associated with a marked increase in systemic inflammation, with augmented circulating levels of proinflammatory cytokines, such as tumor necrosis factor alpha (TNF-α), interleukin (IL)-1β, and IL-6 (125). These trigger signaling pathways that favor PASMCs' contraction and proliferation, increasing ET-1 levels and thromboxane (TXA2)/PGI2 ratio (125). Induction of eNOS in PAECs also occurs, although responsiveness to TXA2 superimposes, explaining the dominant vasoconstrictor response in this context (125). Concurrent hypoxic exposure in these neonates amplifies inflammation-mediated vasoconstriction, further increasing TXA2 synthase (TBXAS) in PAECs and decreasing PGI2. Thus, altered balance of arachidonic-acid metabolite production, associated with hypoxia and inflammatory activation, plays a part in the pathogenesis of PPHN (125).

These observations led to the investigation on what role intrauterine/perinatal exposure to inflammation itself, not only when associated with these conditions, could play in the pathogenesis of PPHN (Figure 6).

Infants with PPHN show increased levels of cytokines, such as IL-1β, TNF-α, IL-6, and IL-8, in tracheal aspirates, demonstrating acute lung inflammation, independently from iNO exposure, underlying primary lung disease or mechanical ventilation (126). This suggests a specific role of inflammation on the pathogenesis of PPHN. Furthermore, the presence of histological chorioamnionitis and/or funisitis, hence perinatal inflammation, is associated with more severe PPHN, with increased requirement of iNO and more advanced respiratory support (127). In addition, term neonates with hypoxic respiratory failure requiring iNO show raised blood levels of proinflammatory mediators in the cord blood, further suggesting that PPHN is mediated by inflammation-induced changes in pulmonary vascular development and tone (128).

This is consistent with the developmental effects of intrauterine inflammation observed in experimental models. Exposure of preterm fetal lambs to intra-amniotic lipopolysaccharide induces chorioamnionitis and alters pulmonary vascular growth proteins, leading to PASMCs hypertrophy (129). Increased vascular remodeling, mediated trough inflammation, adversely alters cardiopulmonary hemodynamics, increasing PAP and PVR and decreasing PBF and cardiac output (129).

There is growing evidence that several perinatal exposures play a role in PPHN. Among those are maternal overweight and diabetes, recognized independent risk factors for PPHN (4). Causality between maternal obesity, diabetes, and PPHN has been suggested to be mediated through inflammation (4).

Maternal high-fat diet, independently or associated with gestational diabetes, has detrimental effects on alveologenesis and vasculogenesis in the developing fetal lung in the rat model (130). This is potentially explained by amplified inflammation, with markedly increased levels of circulating cytokines, leading to alterations in AKT activation, higher ET-1 expression, and impaired VEGF pathway, crucial for vessel development (130). Diabetes and high-fat diet exposed offspring show increased PVR, with echocardiographic evidence of PPHN and higher perinatal death rate (130). These findings support the previous hypothesis by Mayor et al. that HF-diet-induced alterations in fetal lung maturation are mediated by increased placental inflammation and glucocorticoid receptor alterations (131). This further supports that intrauterine/perinatal inflammatory environment alters development and function of the pulmonary vasculature, predisposing to PPHN. This opens the doors for novel therapies to treat PPHN, focusing on modulating inflammation and its mediator cytokines.

#### Potential Targeted Therapies

##### Glucocorticoids

Glucocorticoids (GCs) have potent anti-inflammatory properties and may be beneficial in decreasing cytokine production and function. Some PPHN patients have shown variants in the genes of corticotrophin-releasing hormone receptor 1 and of its binding protein (CRHR1 and CRHBP genes), determining reduced expression of PPAR-γ (109). These genetic abnormalities in the cortisol pathway provide further interest on the potential role of glucocorticoids in the treatment of PPHN.

In PPHN lamb models, antenatal intramuscular betamethasone decreases ET-1 levels and increases SOD2 expression, besides reestablishing eNOS expression and activity, restoring Hsp90–eNOS interactions (132). As such, it reverses increased superoxide and decreased cGMP levels, improving vasodilation in PPHN lambs (132). Moreover, inhaled hydrocortisone significantly improves arterial-to-alveolar ratios and oxygenation (133), besides reducing ROS levels, in part by increased SOD2 activity, and restoring cGMP levels, by normalizing sGC and PDE5 activities and attenuating the deleterious effects of oxidative stress (133). These data propose that GCs may clinically improve oxygenation and decrease hyperoxia-induced alterations in infants as well.

Nevertheless, studies in humans are mostly limited to use of GCs in MAS-associated PPHN (134). However, hydrocortisone has recently shown to significantly increase systolic blood pressure and improve oxygenation in term and near-term infants with PPHN due to all etiologies (excluding congenital abnormalities) and not only MAS (46). However, this was a small retrospective study, involving only 15 infants who received intravenous hydrocortisone as a rescue therapy for severe iNO-resistant PPHN (46). Besides the direct anti-inflammatory effects on pulmonary vasculature, increasing systemic blood pressure may directly contribute to improved oxygenation, since it decreases right-to-left shunting and improves coronary arterial perfusion, thus allowing appropriate pumping of blood from the RV to the lungs (46).

However, appropriate dosing or administration routes of GCs, considering their individual properties and possible side effects, is still largely unknown. The effects of combining GCs and pulmonary vasodilators, although promising, are as well widely unknown (134) and warrant investigation in future studies. Prospective RCTs are required to further evaluate the efficacy and safety of GCs in the treatment of infants with PPHN, in order to eventually support its clinical use.

##### ω-3 Long-chain polyunsaturated fatty acids

Long-chain polyunsaturated fatty acids (LC-PUFAs), such as docosahexaenoic acid, improve the nutritional status and clinical outcomes in septic newborns, attenuating IL-1β response, hence reducing systemic inflammation and organ dysfunction (135). Moreover, ω-3 LC-PUFAs reduce perinatal oxidative stress (136) and attenuate hyperoxia-induced lung injury in newborn rats (137). It has also shown to decrease PVR in lambs, increasing pulmonary artery flow, most likely by competing and decreasing arachidonic acid enzymatic conversion into TXA2, a potent pulmonary vasoconstrictor (67). Thus, a phase 2 clinical trial is set to soon begin recruiting (NCT04031508 http://www.clinicaltrials.gov), aiming to evaluate the effect of a parenteral emulsion containing ω-3 LC-PUFAs on clinical outcomes, inflammation markers and oxidative stress in neonates with PPHN. Thus, this project intends to understand whether the beneficial effects found in septic newborns, besides the experimentally observed decrease on oxidative stress and pulmonary vascular tone, are likewise observed in human neonates with PPHN.

### Serotonin

#### Pathway

Selective serotonin reuptake inhibitors (SSRIs) are known to associate with increased PPHN risk (138). This was the first acknowledged potential link between 5-HT and PPHN and encouraged further investigation on its role on neonatal pulmonary vasculature (Figure 5).

SSRI-exposed neonatal rats develop PPHN, showing increased pulmonary vascular remodeling, RV hypertrophy, decreased oxygenation, and higher mortality at birth (139). SSRI infusion in late-gestation ovine fetus induces pulmonary vasoconstriction and decreases PBF, attributable to increased circulating levels of 5-HT and not to impaired eNOS activity (103). Combined infusion of SSRIs and 5-HT further increases fetal PVR, while ketanserin, a 5-HT_2A_ receptor antagonist, reverses this effect, supporting the observation that 5-HT is a potent fetal pulmonary vasoconstrictor and that its effects are mediated through the 5-HT_2A_ receptor (68, 103). Moreover, ketanserin causes pulmonary vasodilation in normal lamb fetuses, suggesting that endogenous 5-HT contributes to the maintenance of PVR (103), possibly having the same role in humans. Furthermore, 5-HT synthesis is increased in PAECs of PPHN lambs, and both tryptophan hydroxylase (an enzyme that contributes to 5-HT formation) and 5-HT_2A_ receptor expressions are also increased, supporting the role of 5-HT in the pathogenesis of increased pulmonary vascular tone (68).

ROCK inhibition attenuates 5-HT-mediated pulmonary vasoconstriction in the normal lamb, which could indicate that activation of 5-HT_2A_ receptor leads to ROCK activation. However, in PPHN lamb models, fasudil did not reduce 5-HT-induced vasoconstriction, causing vasodilation only when infused alone. As such, the interaction between 5-HT and RhoA/ROCK axes, and its role in the pathogenesis of PPHN, needs further clarification in future studies (68). Besides, activation of 5-HT_2A_ has previously shown to induce activation of other downstream targets, such as generation of ROS and MAP kinase (140). These further interactions might as well be interesting to study in the context of PPHN.

Furthermore, SSRIs could also increase PPHN risk by contributing to premature constriction of the *ductus arteriosus* (DA), a recognized causative factor for PPHN. In fetal mice, SSRIs lead to premature DA constriction *in utero*, while myogenic studies show a concentration-dependent constriction, with diminished sensitivity to PGE1-induced vasodilation, suggesting another potential link between SSRIs and PPHN pathogenesis (141).

#### Potential Targeted Therapies

Endogenous 5-HT likely contributes to the maintenance of fetal PVR and is further increased in experimental PPHN. Moreover, 5-HT_2A_ is the predominant receptor regulating 5-HT-induced perinatal pulmonary vasoconstriction, and its expression is increased in PPHN lamb model (68). As such, modulation of 5-HT signaling may be a rational therapeutic target for the treatment of newborns with PPHN. Blocking 5-HT_2A_ receptor may be useful in the reestablishment of normal PBF. Although ketanserin has experimentally shown to have this effect (68, 103), its use as a therapeutic approach is most likely limited by systemic hypotension, already observed in human adults with PAH (69). Different 5-HT_2A_ antagonists could be another option, such as sarpogrelate, clinically used in human adults for several purposes.

### Other Potential Target Pathways

#### VEGF and Impaired Vascular Growth

Vascular endothelial growth factor (VEGF) plays a prominent role in the normal development of the pulmonary circulation in the fetus and newborn (1). Impaired VEGF signaling may contribute to the pathogenesis of PPHN (Figure 1), since VEGF and its receptor VEGFR are markedly decreased in the lungs of PPHN lambs (142). VEGF levels are also decreased in blood and tracheal aspirates of infants with PPHN (143), further suggesting that decreased VEGF may be likewise involved in human PPHN.

Chronic *in vivo* inhibition of VEGFR has shown to impair vascular growth, increasing PASMC hyperplasia, and to downregulate eNOS, hence inducing PPHN in lamb fetuses (142). As such, VEGF contribution to PPHN is, at least in part, mediated by decreased NO-cGMP signaling (Figure 2), since VEGF increases NO release *in vivo*, leading to pulmonary vasodilation (144).

The mechanisms that link PPHN to decreased arterial growth and reduced alveolarization are still poorly understood, especially when associated with lung hypoplasia, as in CDH (1). These findings show that it may involve altered VEGF–NO signaling. This is particularly relevant since severe PPHN, associated with lung hypoplasia, remains rather iNO resistant, resulting in high mortality (47).

Treatment with recombinant human VEGF (rhVEGF) in PPHN lamb model increases eNOS expression and activity, preserving PAECs' function, and reverses pulmonary vascular remodeling and RV hypertrophy (70).

Further knowledge on the contribution of impaired VEGF signaling to abnormal pulmonary vascular reactivity, remodeling and angiogenesis, and on how to modulate it, may lead to novel treatment strategies for refractory PPHN, especially in the setting of severe lung hypoplasia. However, since the described observations, dating more than 15 years, no further studies on this subject have been found.

#### Nogo-B/NgBR Pathway

Recently, it has been shown that Nogo-B/NgBR pathway, involved in pulmonary vascular development, is altered in fetal lambs with PPHN (145, 146) (Figure 1). Decreased NgBR expression is observed in PAECs and PASMCs of PPHN fetal lambs, contributing to abnormal angiogenesis, mediated through increased levels of ROS (Figure 6). Its knockdown reproduces PPHN phenotype, and its overexpression reverts the abnormalities in both cell types of PPHN lamb models (145, 146).

In PAECs, NgBR overexpression increases both AKT, eNOS activities and SOD2 expression and activity, diminishing oxidative stress in PPHN lamb models (145). In PASMCs, NgBR regulates proliferation by modulating endoplasmic reticulum (ER) stress and ROS formation (146). Increased ROS decrease NgBR expression, further contributing to abnormal PASMC phenotype, while lower NgBR expression further increases ROS formation, leading to PASMCs hyperproliferation (146).

These novel findings have implications on understanding pulmonary defective angiogenesis and artery remodeling in PPHN. Further knowledge on the molecular mechanisms involved in decreased NgBR expression in PPHN may lead to development of new therapeutic approaches to restore normal lung growth in this condition.

#### IGF-1/IGF-1R Signaling

In hypoxia-induced PPHN mice model, insulin-like growth factor-1 (IGF-1), a potent activator of the AKT signaling pathway, contributes to the development of PPHN (147) (Figure 1). Its pulmonary expression is upregulated, hence increasing IGF-1/IGF-1R signaling and resulting in AKT activation, leading to proliferative and antiapoptotic cellular responses, both in PAECs and PASMCs (147).

Furthermore, abnormalities on the IGF-1/IGF-1R axis may be a result of epigenetic dysregulation. IGF-1 regulates the expression of ET-1 in PAECs, while IGF-1 expression *per se* is epigenetically regulated by histone deacetylase (HDAC) (147). Inhibition of HDAC with apicidin reduces hypoxia-induced lung activation of IGF-1/AKT signaling, reversing the consequent pulmonary vascular remodeling and attenuating RV hypertrophy (147). Various factors, such as hypoxia, oxidative stress, and inflammation may alter epigenetic events, triggering abnormal expression of vasoactive factors (148). These experimental data could suggest that a novel HDAC/IGF-1 epigenetic pathway is involved in the development of hypoxia-induced PPHN.

Additionally, knockdown of IGF-1 gene in PASMCs protects against hypoxia-induced pulmonary vascular remodeling, pulmonary hypertension, and RV hypertrophy in neonatal mice (149), which was mirrored by inhibition of its receptor (IGF-1R) (149). The same effects were not observed in adult mice, suggesting that this axis might be specifically important in the developing lung (149).

Thus, targeting the IGF-1/IGF-1R axis, including the HDAC/IGF-1 epigenetic pathway, may have therapeutic benefits in the treatment of hypoxia-induced PPHN. This warrants additional studies on the role of IGF-1 signaling and epigenetics in the pathogenesis of PPHN.

## Conclusion

Recent progress in understanding the pathophysiology of PPHN, reviewed in this article, opens doors to targeted novel therapeutic options, more specific and potentially more effective, particularly relevant for PPHN resistant to current treatment or in settings in which iNO is unavailable. Of those, sildenafil is the most studied in human newborns, and its clinical use is recommended in iNO-resistant infants. Besides, smaller observational studies report clinical benefit of prostaglandins, milrinone, and bosentan as vasodilators in newborns with PPHN, as well as glucocorticoids in the context of augmented inflammation.

Experimental evidence in PPHN lamb models supports the efficacy of all these therapeutic approaches, also supporting potential use of emergent therapies, such as sGC activators/stimulators, l-citrulline, ROCK inhibitors, PPAR-γ agonists, rhSOD, antioxidants (such as BH4 analogs), ω-3 LC-PUFAs, 5-HT_2A_ receptor antagonists, and rhVEGF. In fact, some of these are already used in the treatment of adult PAH, although never tested in newborns.

There is a consistent lack of systematic approach in PPHN clinical research, with many difficulties hindering adequately powered RCTs. Failure of recruitment (due to relatively low incidence of PPHN), ethical considerations (impeding placebo enrollment of critically ill infants), and fast clinical deterioration in iNO-resistant infants (hampering a methodological therapeutic assignment) all contribute to insufficient evidence on the clinical use of potential alternative therapies.

However, efforts in this area should not cease, and future research should focus on investigating clinical efficacy and safety of the previously described current and emergent treatment options for PPHN, allowing translation of experimental findings on the etiopathogenesis of PPHN into clinical practice.

## Author Contributions

SM conceived the idea, has been involved in reviewing the literature and drafting the manuscript, and has given final approval of the version to be published. RA has made substantial contributions to conception and design, has been involved in drafting the manuscript, and has given final approval of the version to be published. AL-M revised the content and gave final approval of the version to be published. CB-S has made substantial contributions to conception and design, has been involved in revising it critically for important intellectual content, and has given final approval of the version to be published. All authors contributed to the article and approved the submitted version.

## Conflict of Interest

The authors declare that the research was conducted in the absence of any commercial or financial relationships that could be construed as a potential conflict of interest.
